# Chestnut Shell Tannins: Effects on Intestinal Inflammation and Dysbiosis in Zebrafish

**DOI:** 10.3390/ani11061538

**Published:** 2021-05-25

**Authors:** Graziella Orso, Mikhail M. Solovyev, Serena Facchiano, Evgeniia Tyrikova, Daniela Sateriale, Elena Kashinskaya, Caterina Pagliarulo, Hossein S. Hoseinifar, Evgeniy Simonov, Ettore Varricchio, Marina Paolucci, Roberta Imperatore

**Affiliations:** 1 Department of Science and Technology (DST), University of Sannio, 82100 Benevento, Italy; graorso@unisannio.it (G.O.); sfacchiano@unisannio.it (S.F.); sateriale@unisannio.it (D.S.); pagliarc@unisannio.it (C.P.); etvarric@unisannio.it (E.V.); rimperatore@unisannio.it (R.I.); 2 Institute of Systematics and Ecology of Animals, Siberian Branch of RAS, 630091 Novosibirsk, Russia; yarmak85@mail.ru (M.M.S.); tyrikovaevgenia@gmail.com (E.T.); elena.kashinskaya@inbox.ru (E.K.); 3 Biological Institute, Tomsk State University, 634050 Tomsk, Russia; 4 Department of Natural Sciences, Novosibirsk State University, 630091 Novosibirsk, Russia; 5 Department of Fisheries, Gorgan University of Agricultural Sciences and Natural Resources, 49138-15739 Gorgan, Iran; hoseinifar@gau.ac.ir; 6 Institute of Environmental and Agricultural Biology (X-BIO), University of Tyumen, 625003 Tyumen, Russia; ev.simonov@gmail.com

**Keywords:** zebrafish, intestinal inflammation, microbiota, polyphenols, prebiotics, cytokines

## Abstract

**Simple Summary:**

With the increase in global population the production of animal proteins becomes increasingly crucial. Aquaculture is the first animal protein supply industry for human consumption. Intensive farming techniques are employed to increase productivity, but these may cause stressful conditions for fish, resulting in impaired growth and poor health conditions. Intestinal inflammation is one of the most common diseases of fish in intensive farming. Intestinal inflammation is usually accompanied by an alteration of the microbiota or dysbiosis. Inflammation and dysbiosis are so tightly intertwined that inflammation may contribute to or result from dysregulation of gut microbiota. Natural substances of plant origin rich in bioactive molecules or more simply phytochemicals, have been proved to be able to reduce inflammation and improve the general health status in various commercially relevant species. In this study, we evaluated the effect of tannins, a class of polyphenols, the most abundant phytochemicals, on intestinal inflammation and microbiota in zebrafish (*Danio rerio*), a small freshwater fish become an attractive biomedicine and aquaculture animal model during the last decades. The zebrafish has been employed in a vast array of studies aiming at investigating the essential processes underlying intestinal inflammation and injury due to its conservative gut morphology and functions. In this study, we administered a diet enriched with chestnut shell extract rich in tannins to a zebrafish model of intestinal inflammation. The treatment ameliorated the damaged intestinal morphophysiology and the microbiota asset. Our results sustain that products of natural origin with low environmental impact and low cost, such as tannins, may help to ease some of the critical issues affecting the aquaculture sector.

**Abstract:**

The aim of the present study was to test the possible ameliorative efficacy of phytochemicals such as tannins on intestinal inflammation and dysbiosis. The effect of a chestnut shell (*Castanea sativa*) extract (CSE) rich in polyphenols, mainly represented by tannins, on k-carrageenan-induced intestinal inflammation in adult zebrafish (*Danio rerio*) was tested in a feeding trial. Intestinal inflammation was induced by 0.1% k-carrageenan added to the diet for 10 days. CSE was administered for 10 days after k-carrageenan induced inflammation. The intestinal morphology and histopathology, cytokine expression, and microbiota were analyzed. The k-carrageenan treatment led to gut lumen expansion, reduction of intestinal folds, and increase of the goblet cells number, accompanied by the upregulation of pro-inflammatory factors (TNFα, COX2) and alteration in the number and ratio of taxonomic groups of bacteria. CSE counteracted the inflammatory status enhancing the growth of health helpful bacteria (*Enterobacteriaceae* and *Pseudomonas*), decreasing the pro-inflammatory factors, and activating the anti-inflammatory cytokine IL-10. In conclusion, CSE acted as a prebiotic on zebrafish gut microbiota, sustaining the use of tannins as food additives to ameliorate the intestinal inflammation. Our results may be relevant for both aquaculture and medical clinic fields.

## 1. Introduction

Tannins are water-soluble phenolic compounds present in a wide variety of plants utilized as food and feed [1]. In the last decade, tannins have been used in animal nutrition raising a great interest in the animal feed sector, due to their promising antioxidant, anti-inflammatory and antibacterial properties [2,3]. Tannins have been employed among the monogastric animals, such as poultry and swine, although contrasting results on animal performance and intestinal health have been reported [4]. Tannins are known to be able to interact with biological systems through the induction of some physiological effects, such as antioxidant, anti-allergy, anti-hypertensive, as well as antimicrobial activities [5,6]. Tannins show “prebiotic-like” effects and promote the growth of lactobacilli and bifidobacteria in in vitro and in vivo models [7]. Recent research has revealed that they can improve the intestinal microbial ecosystem and enhance gut health [3]. Sugiyama et al., (2010) reported the protective effect of the Japanese horse chestnut (*Aesculus turbinate*) extract in methotrexate-induced intestinal injury in rats [8]. The addition of tannin-rich extract from chestnut (*Castanea sativa Mill.*) wood ameliorated the intestinal inflammatory status, antioxidant capacity, barrier function, and improved the intestinal microflora in heat-stressed broilers [9].

However, there is a high number of variables that can influence the biological activity of individual or mixtures of natural compounds. *In silico* methods have been developed to obtain information before conducting the biological tests, to reduce the analysis times and costs [10]. Tannins, as well as other polyphenols, can exert both pro-oxidant and anti-oxidant activities [11,12], probably depending on the concentration used [13]. Thus, it is important to determine the correct kind and dosage of tannins for promoting optimal health conditions.

The intestine is the site of nutrient digestion and absorption, and an important barrier against infectious agents and toxins [14,15]. A multitude of agents can induce intestinal oxidative stress [16], morphological impairment [17] and barrier dysfunction [18,19], resulting in a decreased absorptive function and an increased inflammatory response [20]. Moreover, intestinal inflammation accompanies the proliferation of some potentially pathogenic bacteria, which could exacerbate the barrier impairment and the inflammatory response in the intestine [21,22,23].

Intestinal inflammation is one of the most common diseases of fish in intensive farming. Indeed, the gastrointestinal apparatus, mainly in aquatic animals, is particularly sensitive, being to a more direct contact with the external environment and the possible contaminants. It is the main site of entry of bacterial, viral, and parasitic infections, which represent a major risk for the yield of aquaculture production [24,25]. Moreover, the artificial diet administered during the farming conditions may further stress the gastrointestinal apparatus, with consequent negative effects on health and growth [26,27].

The diets evaluation in aquaculture species requires lengthy trials and high costs [28]. Given its small size, high fecundity, and the full knowledge of its genome, the zebrafish (*Danio rerio*) has become an attractive biomedicine and aquaculture animal model during the last decade [29,30]. Due to the well conserved gut morphology and functions, the zebrafish represents to date an ideal organism to achieve the preliminary evaluation of diets and has been employed in a vast array of studies aiming at investigating the essential processes underlying intestinal inflammation and injury [28,31,32].

Recent studies have shown that chestnut shell tannins improve the immune response of rainbow trout in vitro [33] and the health status, feeding and growth performances of farmed fish Nile tilapia (*Oreocromis niloticus*) [34], beluga sturgeon (*Huso huso*) [35], convict cichlid (*Amatitlania nigrofasciata*) [36] and common carp (*Cyprinus carpio*) [37]. However, no specific studies have been carried out to investigate the potential role of chestnut (*Castanea sativa*) shell tannins in affecting the intestinal inflammation in fish. Thus, we designed this study to investigate the effects of the chestnut shell extract rich in tannins on induced intestinal inflammation in zebrafish (*Danio rerio*), using a multidisciplinary approach: histological, immunohistochemical, molecular and microbiological.

## 2. Materials and Methods

### 2.1. Fish Husbandry

Male adult zebrafish (*Danio rerio*) (weight 0.58 ± 0.04 gr) were employed. Experiments were performed at the Vakili Ornamental fish production complex, with the Permission of the Department of Fisheries, Gorgan University of Agricultural Sciences and Natural Resources, Gorgan, Iran (GUASNR). Animals were acclimated in recirculating tanks at a density of 1 individual L^−1^. Animals were housed under standard conditions of photoperiod (14/10 h of light/dark cicle with Zeitgeber Time-ZT0 at 9 AM (Anti Meridian)) and temperature (28 °C). Animals were fed once per day at 11:00 AM with *Artemia salina*. The feed was provided at 2% of the body weight. During all the experiments, water temperature, pH, nitrites, nitrates, and dissolved oxygen were monitored daily. The water system was a closed circuit with constant aeration and 10% daily exchange. Before the feeding trial, the fish were acclimatized to the laboratory conditions for two weeks. Fish used in this study were treated in accordance with the GUASNR guidelines for animal husbandry. All efforts were made to minimize fish suffering. Zebrafish did not receive medical treatment prior or during the experience. No deaths occurred in the facilities before the euthanasia of animals used for the experiments.

### 2.2. Intestinal Inflammation Induction

In preliminary experiments, adult zebrafish were fed from 3 days up to 4 weeks with peeled *Artemia salina* cysts (22% fat, 44% proteins, 16% carbohydrates: Aqua Schwarz) with and without variable percentages (0.05, 0.1 and 1%) of k-carrageenan (Sigma, St. Louis, MO, USA), a polysaccharide capable to improve immunity parameters and increase growth and survival in cultured aquatic species [38], but known also for its pro-inflammatory effects on the intestine [39,40,41]. The morphological analysis was performed with classic histological techniques on paraffin-embedded samples stained with hematoxylin-eosin (H&E). Goblet cells and leukocytes infiltrates were analyzed with Alcian blue staining. Morphological analysis was carried out at day 3, 10, 14 and 28.

### 2.3. Preparation of Chestnut Shell Extract

Chestnut shells were obtained from Chestnuts (*Castanea sativa* Mill., cultivar ‘‘Palomina’’) grown in Montella (Avellino, Italy). Shells were extracted as previously reported [33]. Briefly, shells were air dried and ground in a domestic grinder. A total of 2 g of shell powder were extracted with 20 mL of water under magnetic stirring for two hours at 75°C. The total phenolic content was determined by the Folin-Ciocalteu colorimetric method [42], modified according to Picariello et al., (2016) [43]. Gallic acid (GA) (Sigma, St. Louis, MO, USA) was used as standard to construct the calibration curves. Results are expressed as mg GA equivalent/g of chestnut shell (mg GAE/g). Samples were assayed in triplicate and values were averaged. The absorbance was monitored at 765 nm.

### 2.4. High Performance Liquid Chromatography (HPLC) Analysis

Polyphenols analysis was performed by LC-4000 Series Integrated HPLC Systems (JASCO, Tokyo, Japan) consisting of a column oven (model CO-2060 plus), set at 30 °C, a UV/Vis photodiode array detector (model MD-2018 plus), an intelligent fluorescence detector (model PF-2020 plus), a liquid chromatography pump (model PU-2089 plus), an autosampler (AS-2059plus) and a ChromNAV software program (JASCO, Japan). A C18 Luna column 5 μm particle size, 25 cm × 3.00 mm I.D. (Phenomenex, Torrance, CA, USA) was used, with a guard cartridge of the same material. All solvents were filtered through a 0.45 μm filter disk (Millipore Co., Bedford, MA, USA For chestnut shell HPLC mobile phase were: water:formic acid (99.80:0.20, *v*/*v*) (A) and methanol (B). Running conditions were: 0 min 95% A and 5% B; 0–45 min 55% B. The system was equilibrated between runs for 10 min using the starting mobile phase composition. The temperature was maintained at 30 °C. Each sample was analyzed three times. The flow-rate was 0.8 mL/min. The injection volume was 20 μL.

### 2.5. Experimental Design and Feeding Protocol

The control diet consisted of peeled *Artemia salina* cysts. The proinflammatory diet consisted of *Artemia salina* mixed with 0.1% of k-carrageenan. The chestnut shell extract (CSE)-enriched diet consisted of *Artemia salina* mixed with CSE. The *Artemia salina* cysts were reduced in powder and then mixed with k-carrageenan or CSE. Water was added until a soft mixture was obtained. The mixture was poured into a dish plate and placed in oven at 37 °C to complete dry. Then the mixture was removed, powdered and stored at RT in a sealed bag. After two weeks of acclimation, the animals were divided into four dietary groups (*n* = 32 fish per group) and treated as follows: (1) control group (C): fed with *Artemia salina* in weight-maintaining amount; (2) inflamed group (I): fed with the same amount of control of *Artemia salina* + 0.1% k-carrageenan for 10 days; (3) treated with CSE group (CSE): fed with *Artemia salina* + CSE (4 µg/d/zebrafish) for 10 days; (4) post-treated group (CSEpostI): fed with *Artemia salina* + 0.1% k- carrageenan for 10 days followed by *Artemia salina* + CSE (4 µg/d/zebrafish) for 10 days. In previous experiments, two levels of inclusion of CSE were chosen and tested: 0.4 and 4 µg/zebrafish. The choice was based on previous studies carried out by the authors of this study and available in the literature [33,34,35,36]. After the trial zebrafish were fasted overnight and sacrificed. For every treatment, intestine from six fish were collected for histological and immunohistochemical analysis, ten intestines for cytokine analysis, six intestines for microbiota by culture dependent methods, and ten intestines for microbiota by metagenomic analysis.

### 2.6. Hystological Analysis

The intestine samples were processed as reported in Varricchio et al., (2012) [44]. Briefly, they were fixed in 4% formalin in 0.01 M phosphate-buffered saline (PBS) pH 7.4 for no longer than 24 h at 4 °C and then dehydrated in a graded series of ethanol and cleared with xylene before being embedded in paraffin. Samples were cut into 5 µm sections with a microtome (Leica Microsystems). Subsequently, anatomically comparable sections of medial intestine (MI) were deparaffinized with xylene and stained with haematoxylin-eosin (H&E) for the morphological analysis. In order to perform a quantitative estimation of the goblet cell number, sections of MI (*n* = 6 animals/treatment; *n* = 3 pairs of sections/animal, each section selected at a 50µm distance to avoid counting the same cells) were stained with Alcian blue (1 g of Alcian blue, pH 2.5, 3 mL/L of acetic acid, and 97 mL of distilled water) for 1 h. Afterwards, the intestine slides were rinsed in tap water for 10 min, oxidized in periodic acid (5 g/L) for 5 min, rinsed in lukewarm tap water for 10 min and dehydrated in Alcohol and clarified in xylol. Histological sections were examined under a Leica DMI6000 light microscopy equipped with Leica DFC340 digital camera (Leica Microsystems) at 10× or 20× magnification. For each section the MI was divided into three regions and goblet cells were counted in each villus of each region. For the score quantification, a total of 10 folds per intestine and 6 intestines were evaluated for each experimental group from three independent experiments. Two independent operators blinded to the experimental protocol analyzed the sections.

### 2.7. Immunohistochemical Analysis

Anatomically comparable sections of MI were deparaffinized and prepared to be stained by the avidin-biotin immunohistochemical technique. Monoclonal antibodies raised in mouse against tumor-necrosis factor-α (TNFα) (code ab1793, Abcam, Cambridge, UK), and polyclonal antibodies raised in rabbit against cyclooxygenase 2 (COX2) (code 69720, NovaTeinBio, Woburn, MA, USA) were employed. The sections were incubated with 0.1% H_2_O_2_ for 5 min to inactivate the endogenous peroxidase activity and then incubated with 10% normal goat serum (NGS) (Vector Laboratories, UK) in 0.1 M Tris-buffered saline, pH 7.6, containing 0.3% Triton X-100 for 30 min. Thereafter, slides were incubated overnight at 4 °C with primary antibodies (1:200 in the NGS). The day after, the sections were rinsed several times and then incubated for 2 h, at room temperature, in biotinylated goat anti-mouse or goat anti-rabbit immunoglobulin with appropriate dilution (Vector Laboratories). Following incubation with secondary antibody, the slides were incubated for 1 h with the avidin-biotin complex diluted in Tris-buffered saline according to the manufacturer’s instructions (ABC Kit; Vectastain, Vector) and then with 0.05% of 3′-diaminobenzidine (DAB) for 10 min (DAB Sigma Fast, Sigma-Aldrich, Milano, Italy). The sections were examined under Leica DMI6000 light microscopy (Leica Microsystems, Wetzlar, Germany) and the digital images were acquired under constant light illumination and magnification (20×), using a digital camera working on gray levels (JCV FC 340FX, Leica). To quantify the density of TNFα- and COX2-positive signal each section of MI was divided into three regions (*n = 6* animals/treatment; *n* = 3 pairs of sections/animal, each section selected at 50 µm distance). Densitometric analysis of TNFα and COX2 peroxidase-based immunostaining was performed by measuring optical density using the image analysis software Image Pro Plus^®^ 6.0 (MediaCybernetics, Rockville, MD, USA) working on a logarithmic scale of absorbance. In each region, the optical density zero value was assigned to the background (i.e., a tissue portion devoid of stained cells) [45,46,47]. All the histological analyses were performed by an independent operator blinded to the type of treatment. The specificity of all the used antibodies was validated with controls as reported in Imperatore et al., 2020 [47].

### 2.8. RNA Isolation, cDNA Synthesis and Real-Time PCR

The mRNA expression levels of *COX-2A, IL-1β, IL-8, IL-10* and *TNFα* in intestinal tissues were detected by quantitative real-time PCR. In order to detect the gene expression, total RNA was extracted from intestinal samples by using Trizol Reagent (Thermo-Scientific, Waltham, MA, USA) according to the manufacturer’s instructions. The RNA purity was determined by electrophoresis in 1% agarose gels and its concentration was determined with a Nanodrop 1000 Spectrophotometer (Thermo-Scientific, Waltham, MA, USA). Total RNA (1 μg) was used to generate cDNA strands in a 20-μL-reaction volume (SensiFAST™ cDNA Synthesis Kit, Bioline). Real-time quantitative RT-PCR was carried out on a QuantStudio 5 System (Thermo Fisher Scientific) using SensiFAST™ SYBR Lo-ROX Kit (Bioline Scientific, Rome, Italy) as a reference dye in a total volume of 20 μL per reaction. The qRT-PCR analysis was performed in triplicate for target mRNAs and the PCR conditions were as follows: 95 °C, 2 min, 40 cycles of 95 °C for 5 s, 60 °C for 10 s and 72 °C for 20 s. Melting curve analysis was performed to confirm specificity. The relative mRNA expression levels of genes were calculated by the 2^−ΔΔCt^ method. Relative quantification of mRNA expression was performed using *β-actin* and *tuba1* as housekeeping genes to standardize results by removing variations in mRNA. *actb1* and *actb2* are two of the most stable and suitable genes for zebrafish intestine [48,49,50,51]. Primers with efficiency close to reference gene were selected. The primer sequences used are listed in Table 1:

### 2.9. Microbiota Intestinal Isolation and Analysis by Culture-Dependent Methods

After sacrifice, intestinal tracts were aseptically removed from fish specimens, maintaining their integrity as much as possible. They were mechanically crushed in sterile test tubes within the appropriate volume of diluent (Buffered Peptone Water, Oxoid, Waltham, MA, USA) necessary for the normalization (1:100 ratio sample/diluent), and were shaken by vortex stirrer, at regular intervals in order to prevent samples overheating, until visible dispersion of the sample in the diluent. Subsequently, serial dilutions were prepared, and an aliquot of each dilution was spread on different culture media. In particular, the researched microorganisms and the microbiological media and incubation conditions used for the analysis were the following: total aerobic mesophilic bacteria on Luria Bertani (LB) Agar (CONDA) in aerobic conditions; *Enterobacteriaceae* on MacConkey Agar (CONDA) in aerobic conditions; *Pseudomonas* spp. on Cetrimide Agar Base (CONDA) in aerobic conditions; *Staphylococcus* spp. on Baird Parker Agar (BPA) (CONDA) in aerobic conditions; total anaerobic bacteria on Tryptose Sulfite syscloserine (TSC) (CONDA) in anaerobiosis; yeasts on Sabouraud Agar (CONDA) in aerobic conditions. Plates were incubated under the appropriate conditions for 24–96 h for microbial growth, in order to make the count of viable colonies. Anaerobiosis was recreated using the AnaeroJar anaerobic jar (BioClass) and the special bags for Atmosphere Generation System CampyGenTM (Thermo Fisher Scientific). Representative microbial isolates, of each group in pure culture, were stored frozen at −80°C in broth media supplemented with 10% glycerol (*v*/*v*) (Carlo Erba Reagents, Waltham, MA, USA). Microbial counts were expressed as the Log CFU g-1 ± SD of triplicates.

### 2.10. Microbiome Analysis

Intestinal samples were used for DNA extraction procedure. Before the DNA extraction, all 40 samples were placed into sterile microcentrifuge tubes with lysis buffer and mechanically homogenized by pestle for 1 min using a handheld Homogenizer. All samples were processed to extract DNA following the DNA-sorb B kit manufacturer’s protocols (kit for DNA extraction, Central Research Institute of Epidemiology, Russia).

### 2.11. 16S rDNA Library Sequencing and Processing

Genomic DNA extracted from the samples was amplified using primer pair (S-D-Bact-0341-b-S17 and S-D-Bact-0785-a-A-21) targeted to the V3–V4 region of the 16S rRNA genes [52]. The forward (5′-TCGTCGGCAGCGTCAGATGTGTATAAGAGA-CAGCCTACGGGNGGCWGCAG-3′) and reverse (5′-GTCTCGTGGGCTCGGAGATGT-GTATAAGAGACAGGACTACHVGGGTATCTAATCC-3′) contained Illumina overhang adapter sequences (underlined regions). Amplicon libraries were subcontracted for preparation and sequencing using an Illumina MiSeq (600 cycles—2 × 300 paired-end) by Evrogen (Moscow, Russia). Nucleotide sequences were deposited in the Sequence Read Archive (NCBI), accession number: PRJNA699145. Read pairs were merged and quality filtered with MOTHUR 1.31.2 [53]. Any reads with ambiguous sites and homopolymers of more than 8 bp were removed, as well as sequences shorter that 350 or greater than 600 bp. QIIME 1.9.1 [54] was used for further processing of the sequences. De novo (abundance based) chimera detection using USEARCH 6.1 [55] was applied to identify possible chimeric sequences (‘identify_chimeric_seqs.py’ with an option ‘-musearch61′ in QIIME). After chimera filtering, the QIIME script ‘pick_open_reference_otus.py’ with default options was used to perform open-reference OTU picking by UCLAST [55], taxonomy assignment (UCLAST, with a 0.80 confidence threshold), sequence alignment (PyNAST1.2.2; [54]) and tree-building (FastTree 2.1.3; [56]). This algorithm involves several steps of both closed-reference and open-reference OTU (Operational Taxonomic Units) picking, followed by taxonomy assignment, where the SILVA reference alignment (release 132) was used as a reference. Chloroplast, mitochondria, and no bacterial sequences were removed from further analysis.

### 2.12. Analysis of Alpha and Beta Diversity

The richness (number of OTU’s and Chao1 index) and diversity estimates (Shannon and Simpson index) per sample were calculated using QIIME. The samples were then rarified to the lowest sequencing effort (4500 sequences) and a weighted UniFrac dissimilarity matrix [57] was calculated in QIIME and used for downstream analysis. To test the effect of k-carrageenan induced inflammation on the groupings of bacterial communities, permutational multivariate analysis of variance (PERMANOVA) using distance matrices were employed as implemented in the ‘adonis’ function of the R-package vegan 2.5-6 [58] (https://CRAN.R-project.org/package=vegan) (accessed on 20 August 2020). Pairwise comparisons for all pairs of levels of used factors were performed using the ‘adonis.pair’ function of EcolUtils 0.1 R-package [59] (https://github.com/GuillemSalazar/EcolUtils) (accessed on 20 August 2020). Analysis of multivariate homogeneity of group dispersions (variances) to test if one or more groups was more variable than the others, was performed using the ‘betadisper’ function of the vegan R-package. In all the aforementioned tests the statistical significance was determined by 10,000 permutations. The matrix was also used to perform principle coordinates analysis (PCoA) to visualize differences among groups of samples. Venn diagrams of OTU membership were calculated using R-package RAM 1.2.1.7 [60] (https://CRAN.R-project.org/package=RAM) (accessed on 13 November 2020). The OTU was designated as a part of core microbiome if OTU was found in more than 50% of samples from each group.

### 2.13. Statistical Analysis

The statistical significance of microbiota data obtained by culture methods was validated by the one-way ANOVA test with Dunnet correction and analyzed with GraphPad Prism 6 software, version 6.05 (GraphPad, Inc., San Diego, CA, USA). One-way analysis of variance with Bonferroni’s post hoc test was adopted for the analysis of normally distributed data. In the case of the experiment concerning densitometric analysis and differences in alpha and beta diversity measures between bacterial groups, the Kruskal–Wallis ANOVA non-parametric test followed by Dunn’s post hoc test was adopted for the analysis of non-normally distributed data. *p*-Values lower than 0.05 were considered statistically significant. The microbiome and densitrometric data are expressed as mean ± SEM and analyzed with GraphPad Prism 6 software, version 6.05 (GraphPad, Inc.).

## 3. Results

### 3.1. Chestnut Shell Extract Composition

In Figure S1 is reported the chromatogram of the chestnut shell extract analyzed by HPLC. Three wavelengths (275, 325, and 375 nm) were chosen in order to detect general phenolic compounds (275 nm), derivatives of hydroxycinnamic acid (325 nm) and flavonols/ellagic acid (375 nm). The only identifiable compound at 275 nm was the Gallic acid, followed by a broad peak formed by condensed tannins [61] that disappeared at 325 and 375 nm.

### 3.2. Intestinal Histopathology

The intestinal inflammation was morphologically visible after three days of feeding with 0.1% of k-carrageenan, while feeding with k-carrageenan at 1% resulted in severe damage of the intestinal villi and disruption of the intestinal tissue integrity (see Figure S2). In the subsequent experiments we employed zebrafish fed for 10 days with *Artemia salina* enriched with 0.1% of k-carrageenan as the inflamed model.

Moreover, since the CSE concentration of 4 µg revealed to be more effective than 0.4 µg on intestinal inflammation resumption, based on H&E and IHC analysis (see Figure S3), this concentration was employed for the subsequent molecular and microbiota analysis.

**Figure 1:**
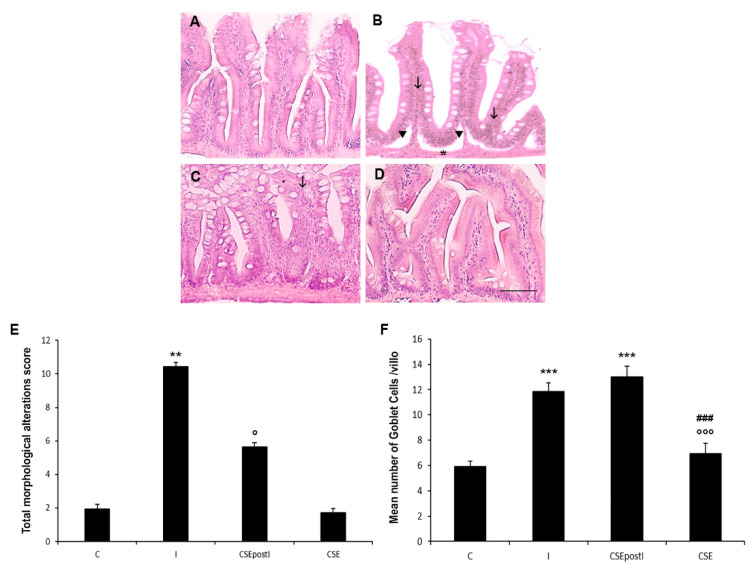
Haematoxylin-eosin (H&E) staining of MI of (**A**) control zebrafish (C), (**B**) inflamed zebrafish (I), (**C**) inflamed zebrafish post-treated with chestnut shell extract (CSEpostI) and (**D**) zebrafish fed chestnut shell extract (CSE). Scale bar: 100 µm. Arrows indicate leucocytes infiltrates, arrowheads indicate ragged villi, asterisks indicate mucosal thinning. (**E**) Bar graph showing the total intestinal alteration score defined for each zebrafish group. Data are expressed as mean ±SE. ** *p* < 0.001 compared to the control group; ° *p* < 0.05 compared to the inflamed group. (**F**) Bar graph showing the number of goblet cells in the MI of C, I, CSEpostI and CSE. Goblet cells were counted based on Alcian Blue staining. Data are expressed as mean ±SE. *** *p* < 0.0001 compared to the control group; °°° *p* < 0.0001 compared to the inflamed group; ### *p* < 0.0001 compared to the CSEpostI group.

The morphological evaluation of the intestine during the feeding experiments is presented in Figure 1 and Figure S4. In order to evaluate the intestinal histological status, we inspired to the score system for histologic evaluation of zebrafish larvae by He et al. [62] but adapted to the adult zebrafish (Table 2, Figure 1). After ten days of feeding with the 0.1% k-carrageenan enriched diet, the intestine showed a significant increase of the score number with respect to the score of the control group, with clear signs of inflammation, such as expansion of gut lumen, as observed by a qualitative analysis, mucosal thinning, ragged, thin and irregular intestinal folds (villi), increase in goblet cell number and leukocyte infiltrates. In particular, the quantitative analysis of histological slides stained with Alcian blue, showed a significant increase in the number of goblet cells/villus in the MI of inflamed zebrafish in comparison to the control (C: 5.9 ± 0.43 vs I: 11,8 ± 0.69; *p* < 0.0001).

CSE treatment partially reverted the morphological alterations, as shown by the significant decrease of the score number compared to the score number of the I group. In particular, CSE restored the intestinal structure, that showed regular and well-organized villi. However, a significantly high number of goblet cells in the MI was present (13 ± 0.84). CSE administered alone did not cause intestinal alterations (Figure 1 and Figure S4) and showed a score number similar to the control. These alterations were also accompanied by a significant increase in the leukocytes infiltrates in the lamina propria and epithelium of both, the I and CSEpostI zebrafish (Figure S4).

**Figure 2:**
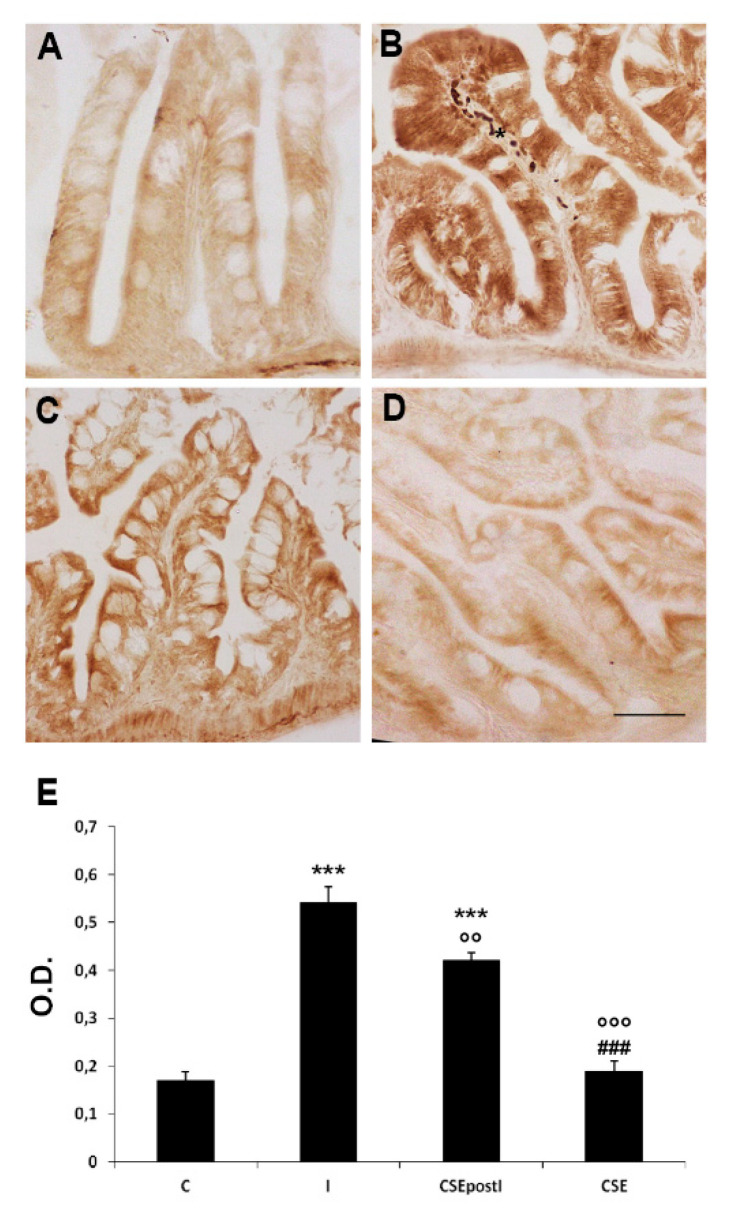
TNFα immunostaining in the MI of (**A**) control zebrafish (C), (**B**) inflamed zebrafish (I), (**C**) inflamed zebrafish post-treated with chestnut shell extract (CSEpostI) and (**D**) zebrafish fed chestnut shell extract (CSE). Scale bar: 50 µm. Asterisks indicate infiltrated eosinophils and fibroblasts expressing TNFα. (**E**) Bar graph showing TNFα optical density (O.D.) in the MI of C, I, CSEpostI and CSE. Data are expressed as mean ±SE. *** *p* < 0.0001 compared to C group; °°° *p* < 0.0001, °° *p* < 0.001 compared to the I group; ^###^ *p* < 0.0001 compared to the CSEpostI group.

**Figure 3:**
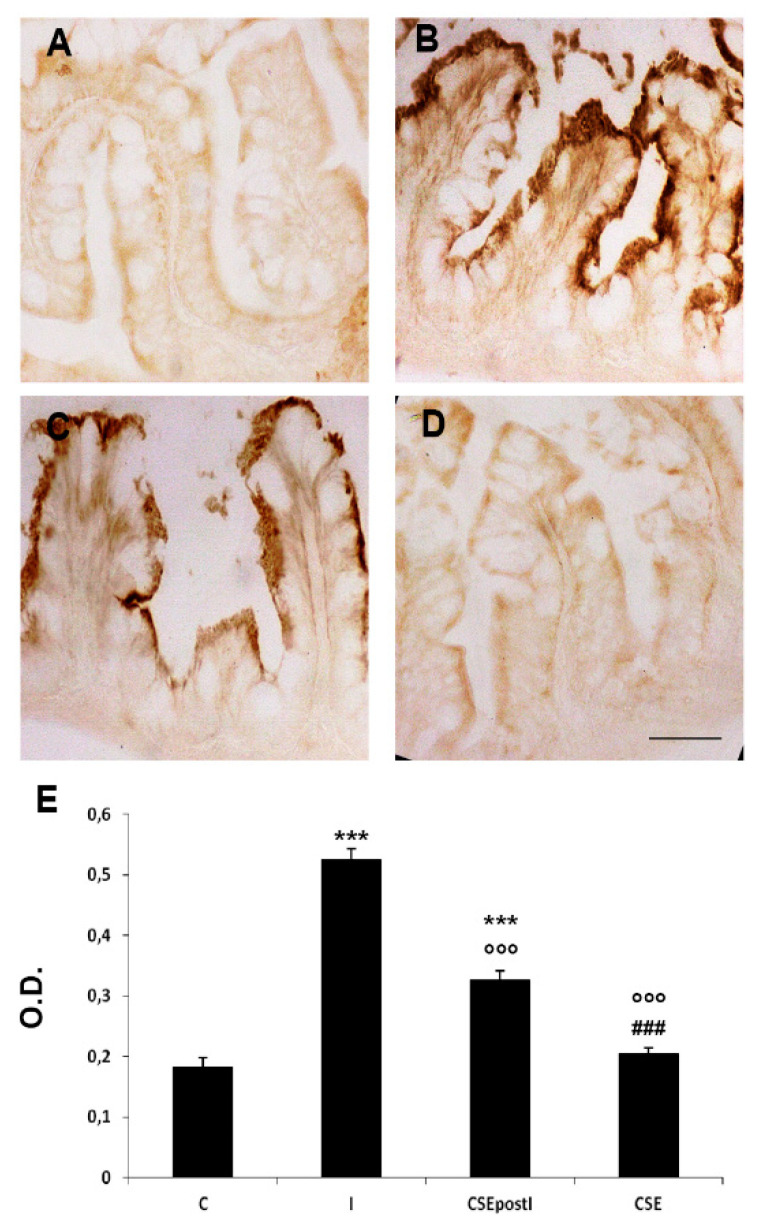
COX2 immunostaining in the MI of (**A**) control zebrafish (C), (**B**) inflamed zebrafish (I), (**C**) inflamed zebrafish post-treated with chestnut shell extract (CSEpostI) and (**D**) zebrafish fed chestnut shell extract (CSE). Scale bar: 50 µm. (**E**) Bar graph showing COX2 optical density (O.D.) in the MI of control, inflamed, CSEpostI and CSE zebrafish. Data are expressed as mean ±SE. *** *p* < 0.0001 compared to control group; °°° *p* < 0.0001 compared to the inflamed group; ### *p* < 0.0001 compared to the CSEpostI group.

Morphological alterations induced by k-carrageenan matched with the increased expression of pro-inflammatory markers, such as TNFα and COX2. In particular, TNFα and COX2 immunoreactivity increased in the enteroendocrine and goblet cells. CSE treatment partially counteracted such increase (Figure 2 and Figure 3). TNFα immunoexpression was detected also in eosinophils and fibroblasts, infiltrated in the lamina propria (Figure 2B). The densitometric analysis showed a significant increase of TNFα optical density in the epithelium of MI of inflamed zebrafish with respect to the control (C: 0.17 ± 0.016 vs I: 0.54 ± 0.03; *p* < 0.0001). However, the post treatment with CSE significantly reduced the TNFα expression with respect to the inflamed zebrafish, although it was significantly higher than the control (I: 0.54 ± 0.03 vs CSEpostI: 0.42 ± 0.02, *p* < 0.001; C: 0.17 ± 0.016 vs CSEpostI: 0.42 ± 0.02, *p* < 0.0001) (Figure 2).

Despite TNFα, COX2 was found largely confined to the villus epithelium. In particular, the epithelial cells of inflamed zebrafish showed a strong immunoreactivity of COX2 on the apical side. The inflammatory status was confirmed by a significant increase of COX2 optical density in inflamed zebrafish with respect to the control (C: 0.18 ± 0.016 vs I: 0.52 ± 0.02, *p* < 0.0001;). CSEpostI zebrafish showed a significant decrease of COX2 expression compared to the inflamed zebrafish (I: 0.52 ± 0.02 vs CSEpostI: 0.39 ± 0.013, *p* < 0.0001), although the expression was significantly higher than the control (C: 0.18 ± 0.016 vs CSEpostI: 0.39 ± 0.013, *p* < 0.0001) (Figure 3).

### 3.3. PCR Analysis

**Figure 4:**
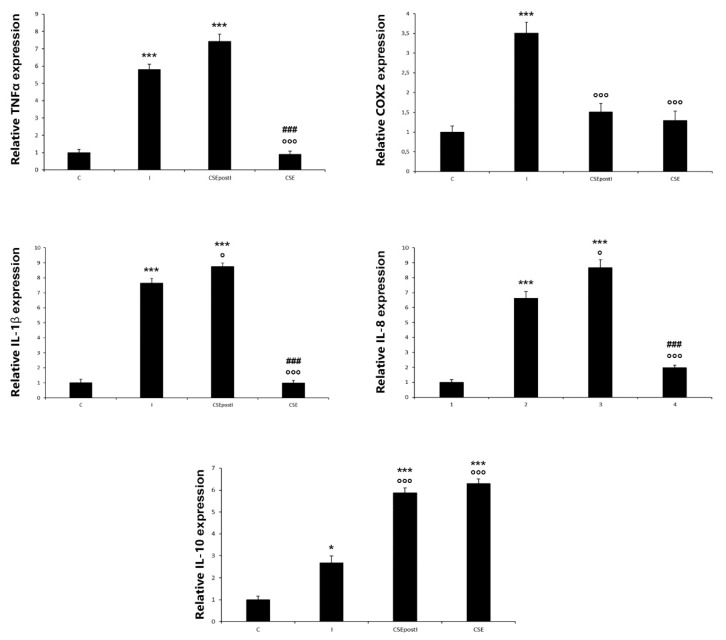
Relative expression of TNFα, COX2, IL-1β, IL-8 and IL-10 genes in control zebrafish (C), inflamed zebrafish (I), inflamed zebrafish post-treated with chestnut shell extract (CSEpostI) and zebrafish fed chestnut shell extract (CSE). Values are presented as the mean ± SEM. n= 6. *** *p* < 0.0001, * *p* < 0.05 compared to control group. °°° *p* < 0.0001, ° *p* < 0.05 compared to the inflamed group. ### *p* < 0.0001 compared to the CSEpostI group.

The quantitative analysis of the relative gene expression of *TNFα*, *COX-2A*, *IL-1β*, *IL-8* and *IL-10* is reported in Figure 4. As detected by optical density, *TNFα* expression was significantly higher in the inflamed (5.79 ± 0.34; *p* < 0.0001) and CSEpostI (7.43 ± 0.42; *p* < 0.0001) zebrafish with respect to the control zebrafish. *COX-2A* expression significantly increased in the inflamed zebrafish (3.51 ± 0.28, *p* < 0.0001) with respect to the control, while it significantly decreased in the CSEpostI (1.51 ± 0.21, *p* < 0.0001) with respect to the inflamed zebrafish. *IL-1β* and *IL-8* expressions were significantly higher in the inflamed zebrafish compared to the control (7.65 ± 0.31 (*p* < 0.0001) and 6.63 ± 0.44 (*p* < 0.0001), respectively). In the CSEpostI zebrafish the relative gene expression levels of the pro-inflammatory cytokines *IL-1β* and *IL-8*, were significantly higher compared to the control group (*IL-1β*: 8.65 ± 0.23, *p* < 0.0001; *IL-8*: 8.67 ± 0.53, *p* < 0.0001). A significant increase was observed for the anti-inflammatory cytokine *IL-10* in CSEpostI (5.86 ± 0.24) and CSE zebrafish (6.3 ± 0.21) (*p* < 0.0001).

### 3.4. Analysis of Intestinal Microbiota by Culture-Dependent Methods

**Figure 5:**
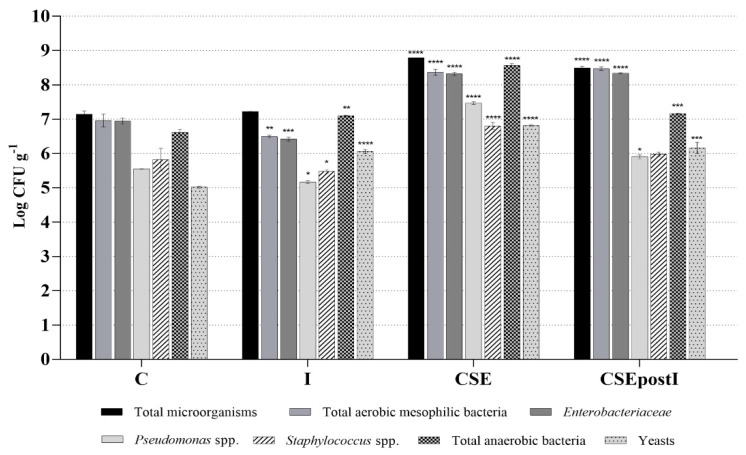
Intestinal microbiota analysis by culture-dependent methods in zebrafish fed on a standard control diet and proinflammatory and polyphenolic diets. The experiments were conducted in triplicate. Results are expressed as Log CFU g-1 and reported as mean ± standard deviation. Asterisks indicate the statistical significance validated by One-Way ANOVA test with Dunnet correction (* *p* < 0.05; ** *p* < 0.01; *** *p* < 0.001; **** *p* < 0.0001), compared to the Control group. The absence of asterisks indicates no significance. C, control group; I, inflamed group; CSE, chestnut shell extract group; CSEpostI, chestnut shell extract administered post inflammation group.

Figure 5 shows the comparison intestinal microbiota profiles of the zebrafish control group, fed on a standard diet, and groups fed on proinflammatory and polyphenolic diets. The average values of triplicate experiments, expressed as Log of colony-forming units (CFU) per gram of intestine, are shown in Table S1. In particular, the amount of total microorganisms in the inflamed group was comparable to the control group, while significantly increased in CSE group, that showed the highest amount of total microorganisms, and in CSEpostI group. Regarding total aerobic mesophilic bacteria, they were slightly reduced in the intestine of I group, but they remarkably increased in the CSE and CSEpostI groups, compared to C group. The amounts of *Enterobacteriaceae* and *Pseudomonas* spp. significantly decreased in the inflamed group, while significantly increased in the CSE group and in the CSEpostI group compared to the control one. A slight decrease of *Staphylococcus* spp. levels was detected in the intestine of I group, while *Staphylococcus* spp. increased in the CSE group and were similar in the control group and in the CSEpostI group. Anaerobic bacteria significantly increased in I group compared to the control; a significant increase was also detected in CSE group and CSEpostI group. Finally, the amount of yeasts significantly increased in I, CSE and CSEpostI groups compared to the control.

### 3.5. Gut Microbiome Metagenomic Analysis

#### 3.5.1. Alpha-Diversity

**Figure 6:**
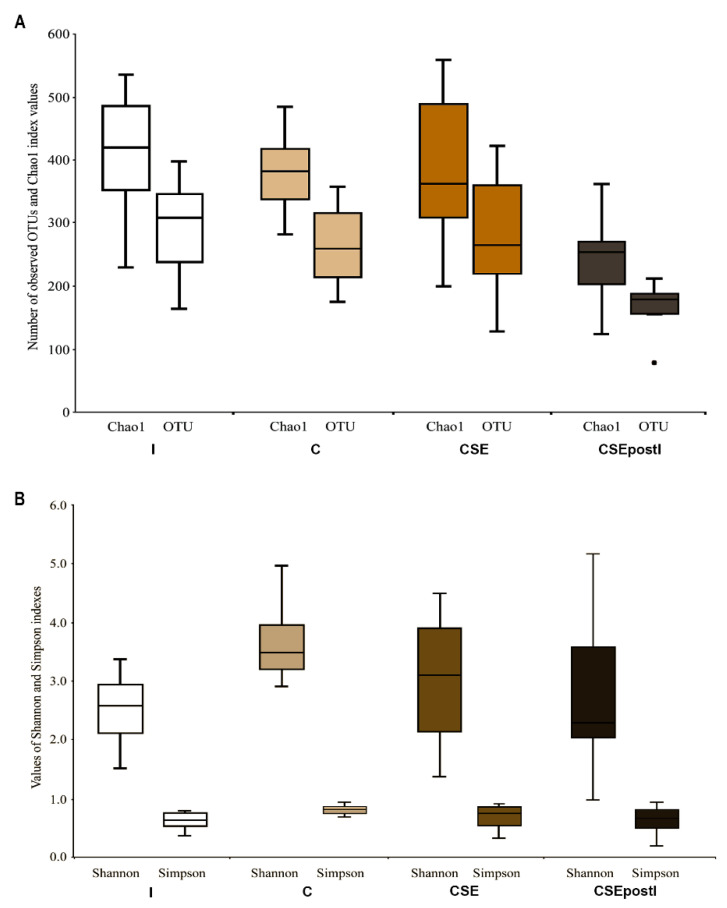
(**A**) Richness (number of observed OTUs and Chao1 index values) and (**B**) diversity (Shannon and Simpson indexes) estimates of bacterial communities from intestine of control zebrafish (C), inflamed zebrafish (I), inflamed zebrafish post-treated with chestnut shell extract (CSEpostI) and zebrafish fed chestnut shell extract (CSE).

The rarefaction curves for all samples are presented in Figure S5. The effect of “group” factor was significant for the number of OTUs (Kruskal–Wallis χ2 test = 16.1, df = 3, *p* = 0.001) and Chao1 ((Kruskal–Wallis χ2 test = 15.6, df = 3, *p* = 0.001) (Figure 6A). The numbers of OTUs and Chao1 index values were significantly lower (*p* ≤ 0.05) in the group CSEPostI (169.0 ± 11.5 and 245.1 ± 20.0, respectevely) than in all other groups (295.2 ± 22.9 and 411.7 ± 29.5; 264.6 ± 17.9 and 380.1 ± 18.2; 281.9 ± 28.4 and 384.2 ± 35.9 for I, control, and CSE, respectively) (Figure 6A). For Simpson and Shannon indexes the effect of “group” factor was significant as well (Kruskal–Wallis χ2 test = 8.8, df = 3, *p* = 0.03 and Kruskal–Wallis χ2 test = 9.7, df = 3, *p* = 0.02, respectively) (Figure 6B). The significantly higher values of Simpson and Shannon indexes were found for the control group (0.81 ± 0.02 and 3.65 ± 0.23, respectively) if compared to the I group (0.65 ± 0.5, Z = −2.77, *p* = 0.017 and 2.5 ± 0.19, Z = −2.73, *p* = 0.019, respectively) and CSEPost I (0.64 ± 0.07, Z = −2.28, *p* = 0.069 and 2.72 ± 0.40, Z = 2.60, *p* = 0.028, respectively). All detailed statistics for alpha-diversity are presented in Table S2.

#### 3.5.2. Beta-Diversity

**Figure 7:**
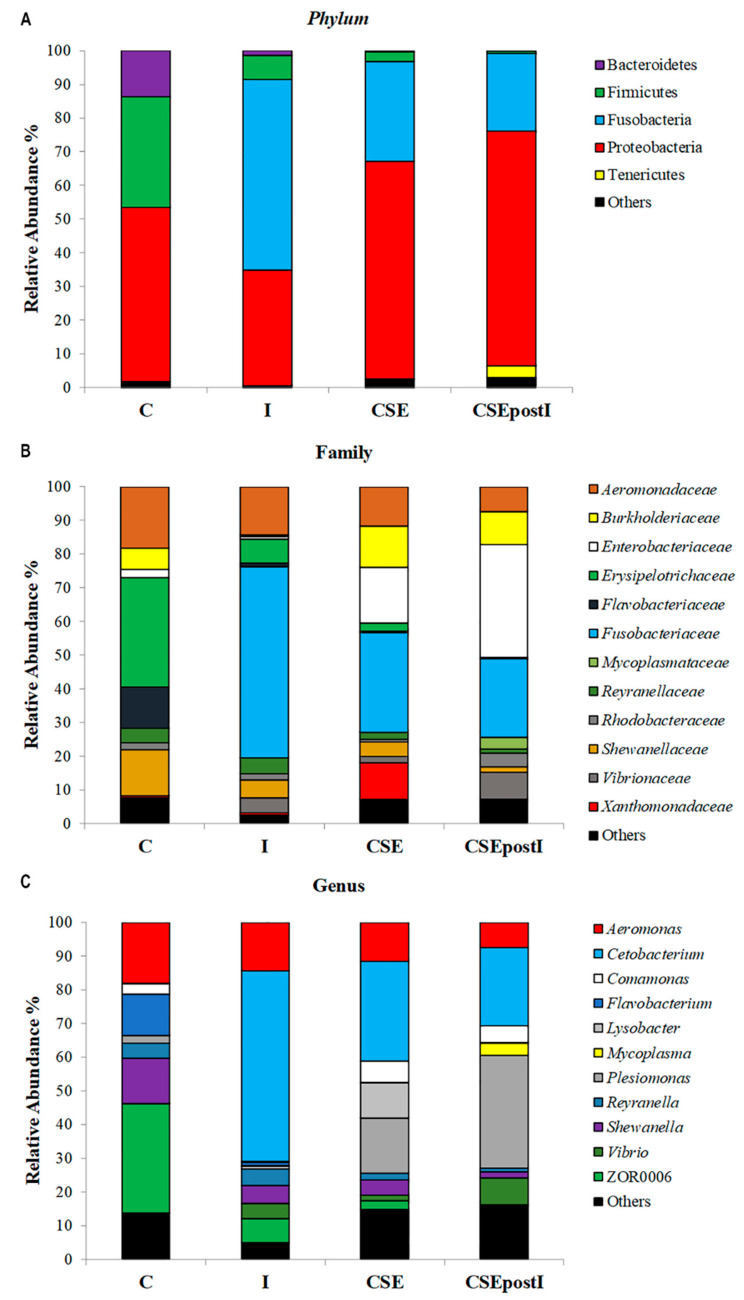
Gut microbiota community composition from intestine of control zebrafish (C), Inflamed zebrafish (I), inflamed zebrafish post-treated with chestnut shell extract (CSEpostI) and zebrafish fed chestnut shell extract (CSE) at phylum (**A**), family (**B**) and genus (**C**) level.

The composition of zebrafish gut microbiome at the phylum level is presented in Figure 7A. In all studied groups, four phyla (Proteobacteria, Firmicutes, Fusobacteria, and Bacteroidetes) represented from 96.9 to 99.5% of all bacterial diversity. In particular, the abundance of the phylum Proteobacteria was significantly higher in the control (51.6 ± 3,9%), CSE (64.6 ± 8.2%), and CSEpostI (69.5 ± 8.1%) groups than in the I group (34.4 ± 4.6%) (PERMANOVA, *p* ≤ 0.05). The abundance of Fusobacteria was significantly higher in the group I (56.6 ± 5.1%) and lower in the control group (0.04 ± 0.02%) (PERMANOVA, *p* ≤ 0.05) if compared to CSE (29.6 ± 8.0%) and CSEpostI (23.2 ± 7.8%) groups, where the difference between them was insignificant (PERMANOVA, *p* = 0.57). The phyla Firmicutes and Bacteroidetes were in relatively high abundance only in the Control group (32.8 ± 4.7% and 13.8 ± 1.4%, respectively). For Bacteroidetes the differences among groups were significant (PERMANOVA, *p* ≤ 0.05). For Firmicutes the differences among groups were significant as well (PERMANOVA, *p* ≤ 0.01) with one exception (CSE vs I, PERMANOVA, *p* = 0.09).

As shown in Figure 7B, from the analysis of family taxonomic level it resulted the presence of: *Aeromonadaceae* (C: 18.1 ± 7.6%; I: 14.4 ± 4.6%; CSE: 11.6 ± 8.5%; CSEpostI: 7.4 ± 7.9%); *Burkholderiaceae* (C: 6.4 ± 14.3%; I: 0.3 ± 0.2%; CSE: 12.2 ± 17.8%; CSEpostI: 9.8 ± 14.8%); *Enterobacteriaceae* (C: 2.3 ± 2.7%; I: 0.9 ± 1.8%; CSE: 16.5 ± 24.6%; CSEpostI: 33.5 ± 29.1%); *Erysipelotrichaceae* (C: 32.5 ± 14.5%; I: 7.1 ± 6.3%; CSE: 2.5 ± 3.1%; CSEpostI: 0.2 ± 0.3%); *Flavobacteriaceae* (C: 12.2 ± 3.8%; I: 1.1 ± 0.1%; CSE: 0.4 ± 0.3%; CSEpostI: 0.1 ± 0.2%); *Fusobacteriaceae* (C: 0.04 ± 0.01%; I: 56.6 ± 15.3%; CSE: 29.6 ± 23,9%; CSEpostI: 23.2 ± 23.3%); *Mycoplasmataceae* (C: 0.00 ± 0.00%; I: 0.04 ± 0.07%; CSE: 0.01 ± 0.03%; CSEpostI: 3.5 ± 9.6%); *Reyranellaceae* (C: 4.4 ± 3.2%; I: 4.7 ± 3.3%; CSE: 1.9 ± 2.6%; CSEpostI: 1.13 ± 2.4%); *Rhodobacteraceae* (C: 2.1 ± 0.7%; I: 1.7 ± 1.2%; CSE: 0.9 ± 1.0%; CSEpostI: 4.1 ± 8.5%); *Shewanellaceae* (C: 13.5 ± 5.2%; I: 5.4 ± 1.7%; CSE: 4.4 ± 3.9%; CSEpostI: 1.8 ± 1.7%); *Vibrionaceae* (C: 0.01 ± 0.02%; I: 4.5 ± 10.7%; CSE: 1.8 ± 4.3%; CSEpostI: 7.9 ± 15.6%); *Xanthomonadaceae* (C: 0.7 ± 1.3%; I: 0.5 ± 0.5%; CSE: 10.7 ± 18.2%; CSEpostI: 0.3 ± 0.5%); Others (C: 7.6 ± 0.05%; I: 2.6 ± 0.01%; CSE: 7.3 ± 0.01%; CSEpostI: 6.9 ± 0.03%).

On a lower taxonomic level (*genus*), the gut microbial community of zebrafish is presented in Figure 7C. We identified eleven bacterial OTUs, the abundance of which was higher than 3.0% at least in one studied group. Genus *Cetobacterium* was highly abundant in all experimental groups (56.6 ± 5.1%, 29.6 ± 8.0%, and 23.2 ± 7.8% for I, CSE, and CSEpostI groups, respectively) whereas in the control group this taxon was almost absent (0.04 ± 0.02%). Similar relationship was found for genus *Vibrio*: the significantly high abundance (PERMANOVA, *p* ≤ 0.05) in the experimental groups (4.5 ± 3.6%, 1.8 ± 1.4%, and 8.0 ± 5.2% for I, CSE, and CSEpostI groups, respectively) if compared to the Control group (0.003 ± 0.002%). In contrast, for ZOR0006, *Shewanella* and *Flavobacterium* significantly higher abundance (PERMANOVA, *p* ≤ 0.05) was found in the control group if compared to the experimental groups (I, CSE, and CSEpostI). For the genera *Aeromonas* and *Reyranella* similar trends were found, with a higher level of abundance in the control group (18.1 ± 2.5% and 4.4 ± 1.1%) if compared to experimental groups (14.4 ± 1.5% and 4.8 ± 1.1%; 11.6 ± 2.8% and 10.3 ± 5.9%; 7.4 ± 2.6% and 1.1±0.8% for the groups I, CSE, and CSEpostI, respectively). For the genera *Comamonas* and *Plesiomonas* similar trends were found: the highest levels of abundance were noted in the groups CSE (6.3 ± 3.1% and 16.5 ± 8.2%, respectively) and CSEPostI (5.1 ± 2.6% and 33.5 ± 9.7%, respectively), then, lower levels were registered in the Control group (3.1 ± 2.6% and 2.3 ± 0.9%, respectively) and the lowest level found in the group I (0.1 ± 0.02% and 0.9 ± 0.6%, respectively). No genus *Mycoplasma* was detected in the control group, whereas in all experimental groups these bacteria were found (0.04 ± 0.02%, 0.01 ± 0.01%, 3.5 ± 3.2% for I, CSE, and CSEPostI respectively). Only the genus *Lysobacter* was clearly dominant in the group CSE if compared to all other groups. All detailed PERMANOVA statistics are given in Table S3.

**Figure 8:**
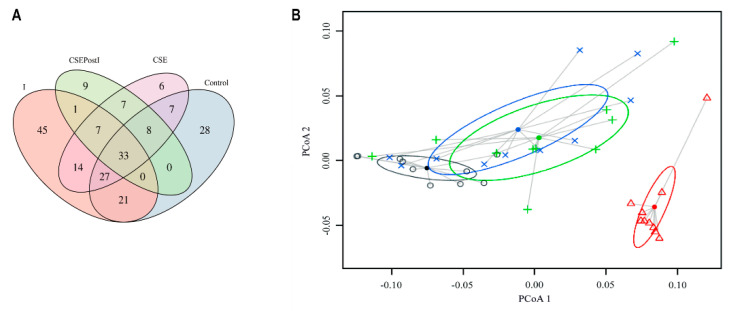
(**A**) Venn diagrams of OTU membership for core microbiota associated with the intestinal content of control zebrafish (C), inflamed zebrafish (I), inflamed zebrafish post-treated with chestnut shell extract (CSEpostI) and zebrafish fed chestnut shell extract (CSE). The OTU was designated as a part of core microbiome if OTU was found in more than 50% of samples from each group. (**B**) Principle coordinates analysis (PCoA) for microbiota associated with the intestinal content of control zebrafish (C), inflamed zebrafish (I), inflamed zebrafish post-treated with chestnut shell extract (CSEpostI) and zebrafish fed chestnut shell extract (CSE). Δ–C; **o**–I; **x**–CSE; +–CSEpostI.

According to Venn diagrams of OTU membership, the core microbiota among the studied groups was represented by 33 OTUs (Figure 8A). The dominant phylum was Proteobacteria (25 out of 33 OTUs) (Table S4). In terms of percentage, it was 12.5% of the content of the control group and 11.2, 11.7, and 19.5% of the content of the I, CSE, and CSEPostI groups, respectively (Figure 8A). A scatter plot based on PCoA scores showed a grouping (ADONIS, F = 13.4, df = 3, *p* = 0.0001) of the microbiota into three groups: (i) control; (ii) CSE and CSEpostI; and (iii) I (Figure 8B).

## 4. Discussion

In the present study, a zebrafish model of intestinal inflammation induced by k-carrageenan addition to the diet was developed to investigate the effects of the chestnut shell extract (CSE) rich in tannins on gut histopathology by morphological analysis, evaluation of cytokines expression and microbiota composition. The data obtained show that k-carrageenan induced intestinal morphological alterations typical of inflammation and increased the expression of pro-inflammatory markers. In particular, inflamed zebrafish were characterized by an increase of the goblet cell number and lymphocytes, accompanied by the enhancement of TNFα and COX2 immunoexpression, and the upregulation of pro-inflammatory cytokines (*IL-1β*, *IL-8*, *TNFα*). This outcome is in agreement with the diet-induced intestinal inflammation characterized by morphological and functional alterations, increase in goblet cells, modifications of villi and alteration of microbiota in common carp (*Cyprinus carpio* L.) [63], in Atlantic salmon (*Salmo salar* L.) [64] and zebrafish [65]. In particular, diet-induced intestinal inflammation in zebrafish is characterized by the increase of pro-inflammatory cytokines, mainly IL-1β, and inflammatory factors, such as TNFα and COX2, accompanied by an increase of neutrophils infiltration, increase in goblet cells, loss of mucosal architecture and villi alteration with fusion of apical cells [65,66].

A number of recent studies proved that k-carrageenan could induce inflammatory events and metabolic disorders in different experimental models, including guinea pigs, mice, rats, monkeys and zebrafish [39,67,68]. Huang et al. [67] showed a sharp increase in leukocyte infiltrates in the intestine of carrageenan-induced inflammation zebrafish model, as soon as 24 h after the intraperitoneal injection of carrageenan, accompanied by an increase of TNFα gene expression in abdominal tissues. Moreover, it has been reported that macroscopic lesions in the cecum and colon of guinea pigs drinking solution of 3% carrageenan for 3 days were accompanied by an increase of macrophages and leukocytes infiltrates. The presence of carrageenan within the epithelial cells associated with mucosa damage was detected [68]. Guinea pigs drinking water supplied with carrageenan for 30 days develop ulcerations in their large intestines showing morphological alteration like epithelial thinning and increase of vacuolated macrophages in the lamina propria accompanied by the typical inflammatory changes [39,40]. A plethora of studies has demonstrated that a chronic carrageenan diet in rat models induces epithelial cell loss, macrophage infiltration, loss of crypts and microscopic mucosal changes in the cecum and/or large intestine [39]. Carrageenan exposure leads to histological changes typical of acute or chronic inflammation in mice, such as mucosa alterations, crypt abscesses, disruption of the epithelial barrier, triggering immune activation and development of inflammatory disease characterized by an increase of IL-6 and TNFα expression and a reduction of IL-10 [69]. Similar inflammatory mechanisms are induced by carrageenan in the human intestine with morphological alteration of mucosa and epithelial barrier, stimulation of pro-inflammatory cytokines and upregulation of TNFα secretion [70]. Therefore, carrageenan is, at date, used to create experimental animal models of inflammation to test the effect of anti-inflammatory drugs and their action on the inflammation mediators and here, for the first time, we performed a k-carrageenan diet-induced inflammation zebrafish model. In line with all these studies, our carrageenan-induced inflammation zebrafish model showed intestinal architecture disruption characterized by an increase of ragged, thin and irregular intestinal folds, expanded gut lumen, increase of goblet cells number and leukocytes infiltrates, upregulation of TNFα, IL-1β and IL-8.

The chestnut shell extract (CSE) is mainly represented by tannins, important anti-inflammatory and antioxidant polyphenolic compounds capable of improving gut health and intestinal microbial ecosystem and for this proposed as an alternative to feed-antibiotics in ruminants and other farm animals [3,71,72].

The diet supplemented with CSE partially counteracted the inflammation, ameliorating intestine morphology, inducing the reorganization of the basal membrane and the thickening of villi, and modulating the expression of some of the cytokines involved in the inflammatory response. Sorice et al. report that phenolic chestnut shell extracts are capable to reduce vascular endothelial growth factor (VEGF) and TNFα in five different human tumor cell lines, suggesting an anti-angiogenic and anti-inflammatory role of CSE [61]. Moreover, a study on the effects of *Dendrobium candidum* polyphenols on a model of zebrafish intestinal inflammation, showed how, in line with our results, polyphenols can counteract the damaged intestinal cells, intestinal mucosal injury, alteration of goblet cells and large number of cytokines (IL-4, IL-10, and TNFα) induced by inflammation [73]. Moreover, polyphenols exert their anti-inflammatory effects, restoring the balance between the expression of pro- (IL-1, TNFα, IL-6, IL-8) and anti-inflammatory cytokines (IL-10, IL-4, TGF), both in vitro and in vivo [74,75,76,77]. Furthermore, polyphenols like resveratrol, curcumin and Epigallocatechin gallate, antagonize inflammation regulating, in vivo and in vitro, not only the expression of the inflammatory cytokines, but also other important inflammatory factors such as cyclooxygenase (COX), lipoxygenase, and prostaglandins in mice and humans [78,79,80,81,82,83]. In line with this, the addition of tannins in the diet of inflamed zebrafish decreased the expression of one of the primary proinflammatory factors, COX2, an important player in the maintenance of the intestinal epithelium, while increased IL-10, one of the most important anti-inflammatory cytokines [84]. These data were consistent with a previous work reporting that polyphenols extracted from olive oil waste water carry out anti-inflammatory activities modulating the gene expression of intestinal COX2 and IL- 10 [85]. Prata et al. [86] showed the anti-inflammatory effect of the medicinal plant *Annona crassilfora* extract in a carrageenan-induced inflammation model of zebrafish. The authors display the capability of natural compound to counteract the morphological intestine alterations, such as damages of villi, lamina propria, epithelial cells and number of goblet cells. Accordingly, our study shows that tannins counteracted, at least in part, the morphological damages induced by k-carrageenan. In particular, tannins were capable of restoring the intestinal structure, leading to regular and well-organized villi, and reporting the gut lumen to normality, even though the number of goblet cells remained high.

The intestine is not only the organ of food absorption and digestion, but also a microbial ecosystem crucial for the intestinal immunity development [87,88,89,90]. The intestinal inflammatory reactions, may cause modifications that disrupt the balance of the intestinal microbiota [91]. Indeed, the complex of inner physic-chemical parameters formed under normal conditions (healthy animal) in host intestine is critically changed during the inflammation process that, consequently, affects the gut microbiota in unpredictable way. In the inflamed intestine the general digestive process and the absorption of nutrients (sugar and peptide) are impaired due to the change in the morphology of the mucosa (villous height and crypt depth) [92]. Trying to heal inflamed tissue, neutrophils release the toxic content of their granules, which include many various components such as reactive oxygen and nitrogen species and hydrolases [93], substances that do not discriminate between non-pathogenic or pathogenic targets, including the microbiota, further damaging the gut [94].

As reported above, in this study, exposure to k-carrageenan caused morphological alteration of the zebrafish intestine compatible with an inflammatory status as well as an alteration of the microbiota. In the gut, the resident microbiota significantly contributes to health maintenance, vitamin and other nutrients synthesis and assimilation, production of short-chain fatty acids, adipose absorption, regulation of host glucose, energy metabolism, development and homeostasis of mucosal and systemic immune system [90,95]. The gut microbiota plays a crucial role in immunity and host response to pathogens in zebrafish [96,97,98]. In zebrafish the gut microbiota is numerically dominated, at all stages of the life cycle, by members of the bacterial phylum Proteobacteria, with the phyla Firmicutes and Fusobacteria also prevalent during larval and adult stages respectively [32,99,100,101]. Fusobacteria are commonly associated with the gastrointestinal tract of freshwater fish and are identified as main contributors to the gut microbiota of fish fed on formulated feed [102]. In our samples Fusobacteria presence was very low. This is in agreement with Chen et al., (2018), who reported the virtual absence of Fusobacteria in the gut microbiota of male and female zebrafish [103]. In zebrafish, despite a core bacterial community, there are variations in relation to the stage of development [100] and dietary changes. In agreement with our feeding conditions based on *Artemia salina*, Chen et al. [103] reported a Proteobacteria-dominated microbiota and absence of Fusobacteria in zebrafish subjected to a diet based on commercial pellets and *Artemia salina*. Further, the microbiome of the gibel carp (*Carassius auratus gibelio*) was similar in larvae fed the same diet [104].

In recent years, it was suggested that the gut microbiome plays a critical role in the development of carrageenan induced inflammation. In mouse, carrageenan alters the microbiota composition inducing a decrease of anti-inflammatory bacteria [89]. Our results highlighted that the administration of proinflammatory diet induces significant changes in the structure of the gut microbiota, in agreement with previous studies that demonstrate how graphene administration induces a condition of dysbiosis in the zebrafish intestine [105].

Metagenomic analysis showed a significant reduction of Proteobacteria in inflamed zebrafish, particularly Gammaproteobacteria, including *Enterobacteriaceae* family and *Shewanella* spp. A similar trend was observed in a study by Gaulke et al. [106], in which adult zebrafish were exposed to a commercial pelletized lab feed causing dysbiosis. Since the majority of *Enterobacteriaceae* in the gut are considered commensals, as they perform beneficial for the host [107,108], and some *Shewanella* species can be considered as fish health modulators due to their probiotics activity [109], the decrease of these bacterial groups can be regarded as a signal of detrimental perturbation in gut microbiome caused by inflammation. The only significant increase among Gammaproteobacteria was observed for *Vibrio*, a common genus in aquatic environments with some species responsible for the most economically important diseases in fish aquaculture [110]. Metagenomic data also showed a reduced abundance for the *phyla* Firmicutes and Bacteroidetes, which are known to be butyrate and short-chain fatty acid producers, respectively, with important roles against gut inflammation [111,112]. The decrease of Firmicutes and Bacteroidetes in inflamed zebrafish, in accordance with other recent studies on dysbiosis of gut microbiota in *Danio rerio* [105,113], was balanced by a notable increase in Fusobacteria, such as *Cetobacterium* spp. Even though the genus *Cetobacterium* has been identified as one of the major components of the microbiota of freshwater fish, such as *Oreochromis niloticus* [114] and *Cyprinus carpio* [115], it has been recently demonstrated that the abundance of *Cetobacterium* in small fishes is negatively correlated with Bacteroides, and this balance might be modulated by the diet [116]. In addition, a remarkable increase in Fusobacteria in zebrafish inflammatory gut model was also reported by Qiao et al. [117].

Increasing evidence is gaining that the health promoting effects attributed to polyphenols derive from the metabolic action of gut bacteria. Indeed, a relevant fraction of polyphenols contained in food is not absorbed in the intestine, but is rather subjected to the action of the microbiota degradation [7]. The metagenomic analysis showed that the treatment of control zebrafish with tannins caused a change in the composition of the microbiota (which sees an increase in both health promoting bacteria and some bacterial groups that increase with inflammation). In particular, the increase in the number of Enterobacteriaceae family was observed also following the stimulating activity of chlorogenic acid and caffeine contained in zebrafish diet [118]. In rats subjected to the diet-inclusion of condensed tannins (proanthocyanidins) extracted from *Acacia angustissima*, an increase in tannin-resistant *Enterobacteriaceae* was reported [119]. However, in some cases the changes induced by tannins seem to resemble those detected in inflamed zebrafish, such as the decrease in the genus *Shewanella* (*phylum* Proteobacteria; class Gammaproteobacteria) and the increase in the genera *Vibrio* (*phylum* Proteobacteria; class Gammaproteobacteria) and *Cetobacterium* (*phylum* Fusobacteria). The similarities with the inflammatory condition are more difficult to explain. It is generally believed that an abnormal intestinal status may result from dysregulation of gut microbiota. However, it is interesting to note that these modifications in microbiota induced by tannins are not accompanied by gut morphological degeneration or cytokine expression compatible to a pathological status. On the contrary, in tannins treated zebrafish the expression of the anti-inflammatory cytokine IL-10 increased, while the expression of the pro-inflammatory cytokins did not increase. So, it is possible to hypothesize that the microbiota deviation from normal is counterbalanced by the dominant abundance of families belonging to the Proteobacteria *phylum*, especially of the helpful *Enterobacteriaceae* family (class Gammaproteobacteria). These results are consistent with recent studies which have demonstrated how Proteobacteria are a microbial signature of inflammation in the gut of mice and human [120,121].

When CSE is administered after inflammation there are some improvements in the microbiota with respect to the picture of inflammatory dysbiosis, although the recovery of the normal intestinal structure is not fully achieved. According to the PCA analysis, the tannins treatment of the inflamed zebrafish shows a microbiota composition very similar to the microbiota of the normal zebrafish treated with tannins. The improvement is also confirmed by the histopathological analysis. The morphology of the tissue appears to be restored, although the proinflammatory cytokines TNFα, IL-8 and IL-1β remain elevated, while COX-2 decreases. Moreover, IL-10 increases. Our results are in agreement with the beneficial effects of polyphenols extracted from *Dendrobium candidum*, an orchid widely used in traditional Chinese medicine, on intestinal inflammation induced by oxazolone in zebrafish [81].

Even if it is widely known that dietary changes can remarkably alter zebrafish gut microbiota, we have focused on the trends of health-promoting and harmful bacteria under different experimental conditions. In particular, both metagenomic and cultural analysis shows that the balance between helpful bacterial groups with respect to potential harmful ones is totally upset in inflamed zebrafish, while it remains preserved in the control group, as well as in groups of zebrafish fed with polyphenols, also when administrated after inflammation. In our study, the changes in intestinal microbiota composition and inflammation regulation might be due to the effect of polyphenols, especially tannins, contained in CSE. Tannins can modulate microbiota, inhibit microbial enzymes or form complex with cell wall components [122]. The chestnut shell extract is dominated by condensed tannins [61], known to induce important changes in the gut microbial community, although it has been reported to exert a low influence on cellulolytic bacteria in zebrafish [81]. This could explain our findings that show only marginal changes in the composition of the microbial community of zebrafish fed on CSE (rich in fibers) with respect to inflamed zebrafish fed with k-carrageenan. The presence of considerable cellulolytic bacterial population, also belonging to Fusobacteria phylum, has been observed in fish digestive tracts [123]. High levels of Fusobacteria were demonstrated in CSE and CSEpostI groups and their absence in control specimens fed only animal protein (*Artemia salina*). The balance between helpful bacterial groups with respect to harmful ones remains evident in the control group, as well as in CSE and CSEpostI groups, where the recovery of the normal condition might be affected by both polyphenolic extracts and microbiota. There is a complex two-way interaction by which polyphenols can modulate gut microbiota and, reciprocally, microorganisms may modulate the activity of polyphenols by regulating their bioavailability and also converting naturally occurring polyphenols into metabolites, which can exert different effects [124]. The dual concept of microbial degradation of polyphenols and modulation of gut microbiota by polyphenols and phenolic metabolites must still be investigated in detail. The literature on the effects of dietary polyphenols on gut microbiota modulation is wide and contradictory. Some studies demonstrated the broad-spectrum antimicrobial activity of polyphenolic extracts also against bacteria belonging to Enterobacteriaceae family [125,126], generally responsible for beneficial effects in fish gut. Moreover, dietary supplementation of polyphenolic extracts at a not adequate concentration may also have adverse effects on beneficial bacteria and thus influence the microbiota balance, which might negatively affect host health and consecuently intestinal morphology [127].

The quantitative and qualitative microbial profiles associated with the zebrafish gut, in the group fed on the control diet and in the groups fed on pro-inflammatory and polyphenolic enriched diets have been studied also by culture-dependent methods, in order to develop fast markers of gut dysbiosis. In particular, results of cultural analysis showed that diet supplemented with k-carrageenan significantly altered the relative composition of the gut bacterial groups by decreasing the abundance of *Enterobacteriaceae* family (*phylum*, Proteobacteria; class, Gammaproteobacteria; order, Enterobacterales) and *Pseudomonas* (*phylum*, Proteobacteria; class Gammaproteobacteria; order, Pseudomonadales; family, *Pseudomonadaceae*) and *Staphylococcus* (*phylum*, Firmicutes; class, Cocchi; order, Coccaceaes; family, *Staphylococcaceae*) genera, and by increasing the levels of anaerobes (such as Fusobacteria), according to the literature data on changes in fish gut microbiota in inflammatory dysbiosis conditions [90,103,117]. For specimens fed on diet supplemented with polyphenols extracted from chestnut shell, the cultural analysis showed a significant increase in the number of *Enterobacteriaceae*, *Pseudomonas* spp. and total anaerobic bacteria (e.g., Lactobacilli and Bifidobacteria), both in zebrafish specimens fed only with polyphenols and in post-treated group. These results are in line with several studies in the literature demonstrating the beneficial effects of phenolic molecules on fishes intestinal microbiota, restoring and improving the fish intestinal health [36,101,118]. So, polyphenols extracted from chestnut shell enhanceed the growth of several health helpful bacteria, such as *Enterobacteriaceae* and *Pseudomonas*. These bacterial groups could constitute possible biomarkers of the health status of the animal host and could constitute potential probiotics for fish health management. For example, the novel strain *P. aeruginosa* FARP72 demonstrated the offering of protection against pathogenic infections in *labeo rohita* [128], validating the hypothesis that the use of probiotics as biocontrol agents for disease prevention may represent an alternative to antibiotics and other drugs, both in aquaculture practices and in medical clinic [129].

Finally, cultural analysis revealed a high yeast content in all experimental groups. Currently, the knowledge of eukaryotic species belonging to the zebrafish intestinal microbiota is poor and inaccurate. The yeast load in the fish gut is variable and can fluctuate from non-detectable levels to up to 10^7^ CFU g^–1^ of intestinal content [130], with *Saccharomyces* spp. and *Rhodotorula* spp. among the most abundant microorganisms [131]. However, this aspect, so far underestimated, needs further study.

## 5. Conclusions

To conclude, this study highlighted how CSE rich in tannins may act as a prebiotic on zebrafish gut microbiota. CSE can improve intestinal health, counteracting intestinal inflammation by regulating immune factors and intestinal microbiota. In particular, tannins exert modulator effects without upsetting the balance between advantageous bacterial groups and potentially harmful groups in the zebrafish gut microbiota, thus allowing the recovery of normobiosis conditions after inflammtory insult. Our work suggests a protective effect of CSE intestinal level, being able to ameliorate the inflammatory status and prevent the progression of intestinal inflammation, making CSE a good candidate as a food additive in farmed fish diets to maintain intestinal health.

## Figures and Tables

**Table 1:** Primer Sequences.

Gene	Forward Primer (5′-3′)	Reverse Primer (5′-3′)
*β-Actin*	TCTCTTAAGTCGACAACCCCC	TCTGAGCCTCATCACCAAG
*COX-2A*	AGGGCGTGTGTTTATCCAAG	ACCTGGACGTCCTTCATAAG
*IL-1* *β*	TGGACTTCGCAGCACAAAATG	GTTCACTTCACGCTCTTGTG
*IL-8*	TGTGTTATTGTTTTCCTGGCATC	GCGACAGCGTGGATCTACG
*IL-10*	CACTGAACGAAAGTTTGCCTAC	TGGAAATGCATCTGGCTTTG
*TNF* *α*	GCTTATGAGCCATGCAGTGA	TGCCCAGTCTGTCTCCTTCT
*tuba1*	CCTGCTGGGAACTGTATTGT	TCAATGAGTTCCTTGCCAAT

**Table 2:** Score system for histologic evaluation.

Score	Intestinal Architecture Disruption		Goblet Cell Number	Leukocyte Infiltrate
	Intestinal Folds	Gut Lumen		
0	Normal	Normal	Normal (~5 GC/villus)	Normal
1	Slight modification	Slight expansion	Increased number (~9 GC/villus)	Appearance of some leukocytes
2	Moderate modification	Moderate expansion	Increased number and different in size (~12 GC/villus)	Increased number of leukocytes in the lamina propria
3	Severe alteration with destruction of the epithelial surface	Severe alterations	Severe high number and morphological changes (~16 GC/villus)	Severe increase of leukocytes in the lamina propria and epithelium

## Data Availability

The raw data supporting the conclusions of this article will be made available by the authors, without undue reservation.

## Supplementary Materials

The following are available online at https://www.mdpi.com/article/10.3390/ani11061538/s1, Figure S1: Chromatographic separation of chestnut shell extract, Figure S2: Hematoxylin-eosin and Alcian Blu staining of MI of control zebrafish and zebrafish fed with and without k-carrageenan, Figure S3: Haematoxylin-eosin staining and TNFα immunostaining of MI of control, inflamed and CSE zebrafish, Figure S4: Alcian blu staining of intestine C, I, CSEpostI and CSE zebrafish, Figure S5: The rarefaction curves for microbiota community from zebrafish intestine, Table S1: Culturable microbiota in zebrafish, Table S2: Pairwise comparisons for richness and diversity estimates, Table S3: Pairwise comparisons among zebrafish groups for genera with the abundance level more than 3%, Table S4: Thirtythree shared bacterial taxons among zebrafish groups of intestinal content.

## References

[1] Serrano J., Puupponen-Pimiä R., Dauer A., Aura A.-M., Calixto F.D.S. (2009). Tannins: Current knowledge of food sources, intake, bioavailability and biological effects. Mol. Nutr. Food Res..

[2] Mueller-Harvey I. (2006). Unravelling the conundrum of tannins in animal nutrition and health. J. Sci. Food Agric..

[3] Huang Q., Liu X., Zhao G., Hu T., Wang Y. (2018). Potential and challenges of tannins as an alternative to in-feed antibiotics for farm animal production. Anim. Nutr..

[4] Caprarulo V., Giromini C., Rossi L. (2021). Review: Chestnut and quebracho tannins in pig nutrition: The effects on performance and intestinal health. Animal.

[5] Sieniawska E. (2015). Activities of Tannins-from in vitro Studies to Clinical Trials. Nat. Prod. Commun..

[6] Smeriglio A., Barreca D., Bellocco E., Trombetta D. (2016). Proanthocyanidins and hydrolysable tannins: Occurrence, dietary intake and pharmacological effects. Br. J. Pharmacol..

[7] Dueñas M., Muñoz-González I., Cueva C., Jiménez-Girón A., Sánchez-Patán F., Santos-Buelga C., Moreno-Arribas M.V., Bartolomé B. (2015). A Survey of Modulation of Gut Microbiota by Dietary Polyphenols. BioMed Res. Int..

[8] Sugiyama A., Kimura H., Ogawa S., Yokota K., Takeuchi T. (2011). Effects of polyphenols from seed shells of Japanese horse chestnut (Aesculus turbinata BLUME) on methotrexate-induced intestinal injury in rats. J. Veter. Med. Sci..

[9] Liu H.W., Li K., Zhao J.S., Deng W. (2017). Effects of chestnut tannins on intestinal morphology, barrier function, pro-inflammatory cytokine expression, microflora and antioxidant capacity in heat-stressed broilers. J. Anim. Physiol. Anim. Nutr..

[10] Rampone S., Pagliarulo C., Marena C., Orsillo A., Iannaccone M., Trionfo C., Sateriale D., Paolucci M. (2021). In silico analysis of the antimicrobial activity of phytochemicals: Towards a technological breakthrough. Comput. Methods Programs Biomed..

[11] Halliwell B. (2008). Are polyphenols antioxidants or pro-oxidants? What do we learn from cell culture and in vivo studies?. Arch. Biochem. Biophys..

[12] Rahal A., Kumar A., Singh V., Yadav B., Tiwari R., Chakraborty S., Dhama K. (2014). Oxidative Stress, Prooxidants, and Antioxidants: The Interplay. BioMed Res. Int..

[13] Prochazkova D., Boušová I., Wilhelmová N. (2011). Antioxidant and prooxidant properties of flavonoids. Fitoterapia.

[14] Vancamelbeke M., Vermeire S. (2017). The intestinal barrier: A fundamental role in health and disease. Expert Rev. Gastroenterol. Hepatol..

[15] Farré R., Fiorani M., Rahiman S.A., Matteoli G. (2020). Intestinal Permeability, Inflammation and the Role of Nutrients. Nutrients.

[16] Gu X., Hao Y., Wang X. (2012). Overexpression of heat shock protein 70 and its relationship to intestine under acute heat stress in broilers: 2. Intestinal oxidative stress. Poult. Sci..

[17] Santos R.R., Awati A., Hil P.J.R.-V.D., Tersteeg-Zijderveld M.H.G., Koolmees P.A., Fink-Gremmels J. (2014). Quantitative histo-morphometric analysis of heat-stress-related damage in the small intestines of broiler chickens. Avian Pathol..

[18] Barreau F., Hugot J. (2014). Intestinal barrier dysfunction triggered by invasive bacteria. Curr. Opin. Microbiol..

[19] Chelakkot C., Ghim J., Ryu S.H. (2018). Mechanisms regulating intestinal barrier integrity and its pathological implications. Exp. Mol. Med..

[20] Quinteiro-Filho W.M., Gomes A.V.S., Pinheiro M.L., Ribeiro A., Ferraz-De-Paula V., Astolfi-Ferreira C.S., Ferreira A.J.P., Palermo-Neto J. (2012). Heat stress impairs performance and induces intestinal inflammation in broiler chickens infected withSalmonellaEnteritidis. Avian Pathol..

[21] Burkholder K.M., Thompson K.L., Einstein M.E., Applegate T., Patterson J.A. (2008). Influence of Stressors on Normal Intestinal Microbiota, Intestinal Morphology, and Susceptibility to Salmonella Enteritidis Colonization in Broilers. Poult. Sci..

[22] Quinteiro-Filho W.M., Ribeiro A., Ferraz-De-Paula V., Pinheiro M.L., Sakai M., Sá L.R.M., Ferreira A.J.P., Palermo-Neto J. (2010). Heat stress impairs performance parameters, induces intestinal injury, and decreases macrophage activity in broiler chickens. Poult. Sci..

[23] Amoroso C., Perillo F., Strati F., Fantini M., Caprioli F., Facciotti F. (2020). The Role of Gut Microbiota Biomodulators on Mucosal Immunity and Intestinal Inflammation. Cells.

[24] Sitjà-Bobadilla A., Estensoro I., Pérez-Sánchez J. (2016). Immunity to gastrointestinal microparasites of fish. Dev. Comp. Immunol..

[25] Vasemägi A., Visse M., Kisand V. (2017). Effect of Environmental Factors and an Emerging Parasitic Disease on Gut Microbiome of Wild Salmonid Fish. mSphere.

[26] Lazzarotto V., Médale F., Larroquet L., Corraze G. (2018). Long-term dietary replacement of fishmeal and fish oil in diets for rainbow trout (Oncorhynchus mykiss): Effects on growth, whole body fatty acids and intestinal and hepatic gene expression. PLoS ONE.

[27] Naylor R.L., Hardy R.W., Bureau D.P., Chiu A., Elliott M., Farrell A.P., Forster I., Gatlin D.M., Goldburg R.J., Hua K. (2009). Feeding aquaculture in an era of finite resources. Proc. Natl. Acad. Sci. USA.

[28] Ulloa P.E., Medrano J.F., Feijoo C.G. (2014). Zebrafish as animal model for aquaculture nutrition research. Front. Genet..

[29] Teame T., Zhang Z., Ran C., Zhang H., Yang Y., Ding Q., Xie M., Gao C., Ye Y., Duan M. (2019). The use of zebrafish (*Danio rerio*) as biomedical models. Anim. Front..

[30] Jørgensen L.V.G. (2020). Zebrafish as a Model for Fish Diseases in Aquaculture. Pathogens.

[31] Brugman S. (2016). The zebrafish as a model to study intestinal inflammation. Dev. Comp. Immunol..

[32] Nadal A.L., Ikeda-Ohtsubo W., Sipkema D., Peggs D., McGurk C., Forlenza M., Wiegertjes G.F., Brugman S. (2020). Feed, Microbiota, and Gut Immunity: Using the Zebrafish Model to Understand Fish Health. Front. Immunol..

[33] Coccia E., Siano F., Volpe M.G., Varricchio E., Eroldogan O.T., Paolucci M. (2019). Chestnut Shell Extract Modulates Immune Parameters in the Rainbow Trout Oncorhynchus mykiss. Fishes.

[34] Van Doan H., Hoseinifar S.H., Hung T.Q., Lumsangkul C., Jaturasitha S., El-Haroun E., Paolucci M. (2020). Dietary inclusion of chestnut (Castanea sativa) polyphenols to Nile tilapia reared in biofloc technology: Impacts on growth, immunity, and disease resistance against Streptococcus agalactiae. Fish. Shellfish. Immunol..

[35] Safari R., Hoseinifar S.H., Imanpour M.R., Mazandarani M., Sanchouli H., Paolucci M. (2020). Effects of dietary polyphenols on mucosal and humoral immune responses, antioxidant defense and growth gene expression in beluga sturgeon (Huso huso). Aquaculture.

[36] Hoseinifar S.H., Jahazi M.A., Nikdehghan N., Van Doan H., Volpe M.G., Paolucci M. (2020). Effects of dietary polyphenols from agricultural by-products on mucosal and humoral immune and antioxidant responses of convict cichlid (Amatitlania nigrofasciata). Aquaculture.

[37] Jahazi M.A., Hoseinifar S.H., Jafari J., Hajimoradloo A., Van Doan H., Paolucci M. (2020). Dietary supplementation of poly-phenols positively affects the innate immune response, oxidative status, and growth performance of common carp, *Cy-prinus carpio* L.. Aquaculture.

[38] Villamil L., Villamil S.I., Rozo G., Rojas J. (2019). Effect of dietary administration of kappa carrageenan extracted from Hypnea musciformis on innate immune response, growth, and survival of Nile tilapia (Oreochromis niloticus). Aquac. Int..

[39] Tobacman J.K. (2001). Review of harmful gastrointestinal effects of carrageenan in animal experiments. Environ. Health Perspect..

[40] Martino J.V., Van Limbergen J., Cahill L.E. (2017). The Role of Carrageenan and Carboxymethylcellulose in the Development of Intestinal Inflammation. Front. Pediatr..

[41] Mohan K., Ravichandran S., Muralisankar T., Uthayakumar V., Chandirasekar R., Seedevi P., Abirami R., Rajan D.K. (2019). Application of marine-derived polysaccharides as immunostimulants in aquaculture: A review of current knowledge and further perspectives. Fish. Shellfish. Immunol..

[42] Singleton V.L., Rossi J.A. (1965). Colorimetry of total phenolics with phosphomolybdic-phosphotungstic acid reagents. Am. J. Enol. Vitic..

[43] Picariello G., De Vito V., Ferranti P., Paolucci M., Volpe M.G. (2016). Species- and cultivar-dependent traits of Prunus avium and Prunus cerasus polyphenols. J. Food Compos. Anal..

[44] Ettore V., Finizia R., Elena C., Giovanni T., David F., Paolo D.G., Marina P. (2012). Immunohistochemical and immunological detection of ghrelin and leptin in rainbow trout Oncorhynchus mykiss and murray cod Maccullochella peelii peelii as affected by different dietary fatty acids. Microsc. Res. Tech..

[45] Imperatore R., Coccia E., D’Angelo L., Varricchio E., De Girolamo P., Paolucci M. (2018). Evidence for leptin receptor immunoreactivity in the gastrointestinal tract and gastric leptin regulation in the rainbow trout (Oncorhynchus mykiss). Ann. Anat. Anat. Anz..

[46] Imperatore R., D’Angelo L., De Girolamo P., Cristino L., Paolucci M. (2019). Identification of Orexin and Endocannabinoid Receptors in Adult Zebrafish Using Immunoperoxidase and Immunofluorescence Methods. J. Vis. Exp..

[47] Imperatore R., Tunisi L., Mavaro I., D’Angelo L., Attanasio C., Safari O., Motlagh H.A., De Girolamo P., Cristino L., Varricchio E. (2020). Immunohistochemical Analysis of Intestinal and Central Nervous System Morphology in an Obese Animal Model (*Danio rerio*) Treated with 3,5-T2: A Possible Farm Management Practice?. Animals.

[48] Jin Y., Xia J., Pan Z., Yang J., Wang W., Fu Z. (2018). Polystyrene microplastics induce microbiota dysbiosis and inflammation in the gut of adult zebrafish. Environ. Pollut..

[49] Rassier G.T., Silveira T.L.R., Remião M.H., Daneluz L.O., Martins A.W.S., Dellagostin E.N., Ortiz H.G., Domingues W.B., Komninou E.R., Kütter M.T. (2020). Evaluation of qPCR reference genes in GH-overexpressing transgenic zebrafish (*Danio rerio*). Sci. Rep..

[50] Luo T., Wang X., Jin Y. (2021). Low concentrations of imidacloprid exposure induced gut toxicity in adult zebrafish (*Danio rerio*). Comp. Biochem. Physiol. Part. C Toxicol. Pharmacol..

[51] Cirmi S., Randazzo B., Russo C., Musumeci L., Maugeri A., Montalbano G., Guerrera M.C., Lombardo G.E., Levanti M. (2020). Anti-inflammatory effect of a flavonoid-rich extract of orange juice in adult zebrafish subjected toVibrio anguillarum-induced enteritis. Nat. Prod. Res..

[52] Klindworth A., Pruesse E., Schweer T., Peplies J., Quast C., Horn M., Glöckner F.O. (2012). Evaluation of general 16S ribosomal RNA gene PCR primers for classical and next-generation sequencing-based diversity studies. Nucleic Acids Res..

[53] Schloss P.D., Westcott S.L., Ryabin T., Hall J.R., Hartmann M., Hollister E.B., Lesniewski R.A., Oakley B.B., Parks D.H., Robinson C.J. (2009). Introducing mothur: Open-Source, Platform-Independent, Community-Supported Software for Describing and Comparing Microbial Communities. Appl. Environ. Microbiol..

[54] Caporaso J.G., Kuczynski J., Stombaugh J., Bittinger K., Bushman F.D., Costello E.K., Fierer N., Peña A.G., Goodrich J.K., Gordon J.I. (2010). QIIME Allows Analysis of High-Throughput Community Sequencing data. Nat. Methods.

[55] Edgar R.C. (2010). Search and clustering orders of magnitude faster than BLAST. Bioinformatics.

[56] Price M.N., Dehal P.S., Arkin A.P. (2010). FastTree 2 Approximately Maximum-Likelihood Trees for Large Alignments. PLoS ONE.

[57] Lozupone C., Knight R. (2005). UniFrac: A New Phylogenetic Method for Comparing Microbial Communities. Appl. Environ. Microbiol..

[58] Oksanen J., Blanchet F.G., Friendly M., Kindt R., Legendre P., McGlinn D., Minchin P.R., O’Hara R.B., Simspon G.L., Solymos P. (2019). Vegan: Community Ecology Package. R Package Version 2.5-4. https://CRAN.R-project.org/package=vegan..

[59] Salazar G. EcolUtils: Utilities for community ecology analysis. R package version 0.1. 2018.

[60] Chen W., Simpson J., Levesque C.A. (2018). RAM: R for Amplicon-Sequencing-Based Microbial-Ecology. R package version 1.2.1.7. https://CRAN.R-project.org/package=RAM.

[61] Sorice A., Siano F., Capone F., Guerriero E., Picariello G., Budillon A., Ciliberto G., Paolucci M., Costantini S., Volpe M.G. (2016). Potential Anticancer Effects of Polyphenols from Chestnut Shell Extracts: Modulation of Cell Growth, and Cytokinomic and Metabolomic Profiles. Molecules.

[62] He Q., Wang L., Wang F., Wang C., Tang C., Li Q., Li J., Zhao Q. (2013). Microbial fingerprinting detects intestinal microbiota dysbiosis in Zebrafish models with chemically-induced enterocolitis. BMC Microbiol..

[63] Urán P., Gonçalves A., Taverne-Thiele J., Schrama J., Verreth J., Rombout J. (2008). Soybean meal induces intestinal inflammation in common carp (Cyprinus carpio L.). Fish. Shellfish. Immunol..

[64] Krogdahl Å., Bakke-McKellep A., Baeverfjord G. (2003). Effects of graded levels of standard soybean meal on intestinal structure, mucosal enzyme activities, and pancreatic response in Atlantic salmon (*Salmo salar* L.). Aquac. Nutr..

[65] Arias-Jayo N., Abecia L., Alonso-Sáez L., Ramirez-Garcia A., Rodriguez A., Pardo M.A. (2018). High-Fat Diet Consumption Induces Microbiota Dysbiosis and Intestinal Inflammation in Zebrafish. Microb. Ecol..

[66] Fehrmann-Cartes K., Coronado M., Hernández A., Allende M., Feijoo C. (2019). Anti-inflammatory effects of aloe vera on soy meal-induced intestinal inflammation in zebrafish. Fish. Shellfish. Immunol..

[67] Huang S.-Y., Feng C.-W., Hung H.-C., Chakraborty C., Chen C.-H., Chen W.-F., Jean Y.-H., Wang H.-M.D., Sung C.-S., Sun Y.-M. (2014). A Novel Zebrafish Model to Provide Mechanistic Insights into the Inflammatory Events in Carrageenan-Induced Abdominal Edema. PLoS ONE.

[68] Ekambaram S.P., Perumal S.S., Pavadai S. (2017). Anti-inflammatory effect of Naravelia zeylanica DC via suppression of inflammatory mediators in carrageenan-induced abdominal oedema in zebrafish model. Inflammopharmacology.

[69] Marcus S.N., Marcus A.J., Marcus R., Ewen S.W., Watt J. (1992). The pre-ulcerative phase of carrageenan-induced colonic ulceration in the guinea-pig. Int. J. Exp. Pathol..

[70] Wei W., Feng W., Xin G., Tingting N., Zhanghe Z., Haimin C., Xiaojun Y. (2016). Enhanced effect of κ-carrageenan on TNBS-induced inflammation in mice. Int. Immunopharmacol..

[71] Borthakur A., Bhattacharyya S., Dudeja P.K., Tobacman J.K. (2007). Carrageenan induces interleukin-8 production through distinct Bcl10 pathway in normal human colonic epithelial cells. Am. J. Physiol. Liver Physiol..

[72] Park M., Cho H., Jung H., Lee H., Hwang K.T. (2013). Antioxidant and Anti-Inflammatory Activities of Tannin Fraction of the Extract from Black Raspberry Seeds Compared to Grape Seeds. J. Food Biochem..

[73] Fumagalli M., SanGiovanni E., Vrhovsek U., Piazza S., Colombo E., Gasperotti M., Mattivi F., De Fabiani E., Dell’Agli M. (2016). Strawberry tannins inhibit IL-8 secretion in a cell model of gastric inflammation. Pharmacol. Res..

[74] Gong X., Jiang S., Tian H., Xiang D., Zhang J. (2020). Polyphenols in the Fermentation Liquid of Dendrobium candidum Relieve Intestinal Inflammation in Zebrafish Through the Intestinal Microbiome-Mediated Immune Response. Front. Immunol..

[75] Comalada M., Ballester I., Bailón E., Sierra S., Xaus J., Gálvez J., de Medina F.S., Zarzuelo A. (2006). Inhibition of pro-inflammatory markers in primary bone marrow-derived mouse macrophages by naturally occurring flavonoids: Analysis of the structure–activity relationship. Biochem. Pharmacol..

[76] Boshtam M., Asgary S., Kouhpayeh S., Shariati L., Khanahmad H. (2017). Aptamers Against Pro- and Anti-Inflammatory Cytokines: A Review. Inflammatory.

[77] Pessina A., Di Vincenzo M., Maradonna F., Marchegiani F., Olivieri F., Randazzo B., Gioacchini G., Carnevali O. (2021). Polydatin Beneficial Effects in Zebrafish Larvae Undergoing Multiple Stress Types. Int. J. Environ. Res. Public Heal..

[78] Yahfoufi N., Alsadi N., Jambi M., Matar C. (2018). The Immunomodulatory and Anti-Inflammatory Role of Polyphenols. Nutrients.

[79] Kim H.P., Son K.H., Chang H.W., Kang S.S. (2004). Anti-inflammatory Plant Flavonoids and Cellular Action Mechanisms. J. Pharmacol. Sci..

[80] Yoon J.-H., Baek S.J. (2005). Molecular Targets of Dietary Polyphenols with Anti-inflammatory Properties. Yonsei Med. J..

[81] Santangelo C., Varì R., Scazzocchio B., Di Benedetto R., Filesi C., Masella R. (2007). Polyphenols, intracellular signalling and inflammation. Ann. Ist. Super Sanita.

[82] Malireddy S., Kotha S.R., Secor J.D., Gurney T.O., Abbott J.L., Maulik G., Maddipati K.R., Parinandi N.L. (2012). Phytochemical Antioxidants Modulate Mammalian Cellular Epigenome: Implications in Health and Disease. Antioxid. Redox Signal..

[83] Hussain T., Tan B., Yin Y., Blachier F., Tossou M.C.B., Rahu N. (2016). Oxidative Stress and Inflammation: What Polyphenols Can Do for Us?. Oxid. Med. Cell. Longev..

[84] Grishin A.V., Wang J., Potoka D.A., Hackam D.J., Upperman J.S., Boyle P., Zamora R., Ford H.R. (2005). Lipopolysaccharide Induces Cyclooxygenase-2 in Intestinal Epithelium via a Noncanonical p38 MAPK Pathway. J. Immunol..

[85] Cárdeno A., Sánchez-Hidalgo M., Alarcón-De-La-Lastra C. (2013). An up-date of olive oil phenols in inflammation and cancer: Molecular mechanisms and clinical implications. Curr. Med. Chem..

[86] Prata M., Charlie-Silva I., Gomes J., Barra A., Berg B., Paiva I., Melo D., Klein A., Romero M.C., Oliveira C. (2020). Anti-inflammatory and immune properties of the peltatoside, isolated from the leaves of Annona crassiflora Mart., in a new experimental model zebrafish. Fish. Shellfish. Immunol..

[87] Mestecky J., Russell M., Elson C.O. (1999). Intestinal IgA: Novel views on its function in the defence of the largest mucosal surface. Gut.

[88] Ouwehand A., Isolauri E., Salminen S. (2002). The role of the intestinal microflora for the development of the immune system in early childhood. Eur. J. Nutr..

[89] Preidis G.A., Ajami N.J., Wong M.C., Bessard B.C., Conner M.E., Petrosino J.F. (2015). Composition and function of the un-dernourished neonatal mouse intestinal microbiome. J. Nutr. Biochem..

[90] Xia J., Lu L., Jin C., Wang S., Zhou J., Ni Y., Fu Z., Jin Y. (2018). Effects of short term lead exposure on gut microbiota and hepatic metabolism in adult zebrafish. Comp. Biochem. Physiol. Part. C Toxicol. Pharmacol..

[91] Da Silva L.M.R., Lima J.D.S.S., Magalhães F.E.A., Campos A.R., De Araújo J.I.F., Batista F.L.A., De Araújo S.M.B., De Sousa P.H.M., Lima G.C., Holanda D.K.R. (2020). Graviola Fruit Bar Added Acerola By-Product Extract Protects Against Inflammation and Nociception in Adult Zebrafish (*Danio rerio*). J. Med. Food.

[92] Peuhkuri K. (2010). Even low-grade inflammation impacts on small intestinal function. World J. Gastroenterol..

[93] Nathan C. (2006). Neutrophils and immunity: Challenges and opportunities. Nat. Rev. Immunol..

[94] Nathan C. (2002). Points of control in inflammation. Nat. Cell Biol..

[95] Webb C.R., Koboziev I., Furr K.L., Grisham M.B. (2016). Protective and pro-inflammatory roles of intestinal bacteria. Pathophysiology.

[96] Bates J.M., Akerlund J., Mittge E., Guillemin K. (2007). Intestinal Alkaline Phosphatase Detoxifies Lipopolysaccharide and Prevents Inflammation in Zebrafish in Response to the Gut Microbiota. Cell Host Microbe.

[97] Galindo-Villegas J., Garcia-Moreno D., De Oliveira S., Meseguer J., Mulero V. (2012). Regulation of immunity and disease resistance by commensal microbes and chromatin modifications during zebrafish development. Proc. Natl. Acad. Sci. USA.

[98] Fawley J., Koehler S., Cabrera S., Lam V., Fredrich K., Hessner M., Salzman N., Gourlay D. (2017). Intestinal alkaline phosphatase deficiency leads to dysbiosis and bacterial translocation in the newborn intestine. J. Surg. Res..

[99] Roeselers G., Mittge E.K., Stephens W.Z., Parichy D.M., Cavanaugh C.M., Guillemin K., Rawls J.F. (2011). Evidence for a core gut microbiota in the zebrafish. ISME J..

[100] Stephens W.Z., Burns A.R., Stagaman K., Wong S., Rawls J.F., Guillemin K., Bohannan B.J.M. (2016). The composition of the zebrafish intestinal microbial community varies across development. ISME J..

[101] Cholan P.M., Han A., Woodie B.R., Watchon M., Kurz A.R., Laird A.S., Britton W.J., Ye L., Holmes Z.C., McCann J.R. (2020). Conserved anti-inflammatory effects and sensing of butyrate in zebrafish. Gut Microbes.

[102] Walburn J.W., Wemheuer B., Thomas T., Copeland E., O’Connor W., Booth M., Fielder S., Egan S. (2019). Diet and diet-associated bacteria shape early microbiome development in Yellowtail Kingfish (Seriola lalandi). Microb. Biotechnol..

[103] Chen L., Zhang W., Hua J., Hu C., Lai N.L.-S., Qian P.-Y., Lam P.K.S., Lam J.C.W., Zhou B. (2018). Dysregulation of Intestinal Health by Environmental Pollutants: Involvement of the Estrogen Receptor and Aryl Hydrocarbon Receptor. Environ. Sci. Technol..

[104] Li X., Zhou L., Yu Y., Ni J., Xu W., Yan Q. (2017). Composition of Gut Microbiota in the Gibel Carp (Carassius auratus gibelio) Varies with Host Development. Microb. Ecol..

[105] Zheng M., Lu J., Lin G., Su H., Sun J., Luan T. (2019). Dysbiosis of gut microbiota by dietary exposure of three graphene-family materials in zebrafish (*Danio rerio*). Environ. Pollut..

[106] Gaulke C.A., Barton C.L., Proffitt S., Tanguay R.L., Sharpton T.J. (2016). Triclosan Exposure Is Associated with Rapid Restructuring of the Microbiome in Adult Zebrafish. PLoS ONE.

[107] Gu W., Tong P., Liu C., Wang W., Lu C., Han Y., Sun X., Kuang D.X., Li N., Dai J. (2019). The characteristics of gut microbiota and commensal Enterobacteriaceae isolates in tree shrew (Tupaia belangeri). BMC Microbiol..

[108] Martinson J.N.V., Pinkham N.V., Peters G.W., Cho H., Heng J., Rauch M., Broadaway S.C., Walk S.T. (2019). Rethinking gut microbiome residency and the Enterobacteriaceae in healthy human adults. ISME J..

[109] Cámara-Ruiz M., Balebona M.C., Moriñigo M.Á., Esteban M.Á. (2020). Probiotic Shewanella putrefaciens (SpPdp11) as a Fish Health Modulator: A Review. Microorganism.

[110] Toranzo A.E., Magariños B., Romalde J.L. (2005). A review of the main bacterial fish diseases in mariculture systems. Aquaculture.

[111] Besten G.D., van Eunen K., Groen A.K., Venema K., Reijngoud D.-J., Bakker B.M. (2013). The role of short-chain fatty acids in the interplay between diet, gut microbiota, and host energy metabolism. J. Lipid Res..

[112] Venegas D.P., De La Fuente M.K., Landskron G., González M.J., Quera R., Dijkstra G., Harmsen H.J.M., Faber K.N., Hermoso M.A. (2019). Short Chain Fatty Acids (SCFAs)-Mediated Gut Epithelial and Immune Regulation and Its Relevance for Inflammatory Bowel Diseases. Front. Immunol..

[113] Jin C., Luo T., Zhu Z., Pan Z., Yang J., Wang W., Fu Z., Jin Y. (2017). Imazalil exposure induces gut microbiota dysbiosis and hepatic metabolism disorder in zebrafish. Comp. Biochem. Physiol. Part. C Toxicol. Pharmacol..

[114] Tsuchiya C., Sakata T., Sugita H. (2007). Novel ecological niche of Cetobacterium somerae, an anaerobic bacterium in the intestinal tracts of freshwater fish. Lett. Appl. Microbiol..

[115] Van Kessel M.A., Dutilh B.E., Neveling K., Kwint M.P., Veltman A.J., Flik G., Jetten M.S., Klaren P.H., Camp H.J.O.D. (2011). Pyrosequencing of 16S rRNA gene amplicons to study the microbiota in the gastrointestinal tract of carp (*Cyprinus carpio* L.). AMB Express.

[116] Hao Y.T., Wu S.G., Xiong F., Tran N.T., Jakovlić I., Zou H., Li W.X., Wang G.T. (2017). Succession and Fermentation Products of Grass Carp (Ctenopharyngodon idellus) Hindgut Microbiota in Response to an Extreme Dietary Shift. Front. Microbiol..

[117] Qiao R., Deng Y., Zhang S., Wolosker M.B., Zhu Q., Ren H., Zhang Y. (2019). Accumulation of different shapes of microplastics initiates intestinal injury and gut microbiota dysbiosis in the gut of zebrafish. Chemosphere.

[118] Osimani A., Milanović V., Roncolini A., Riolo P., Ruschioni S., Isidoro N., Loreto N., Franciosi E., Tuohy K., Olivotto I. (2019). Hermetia illucens in diets for zebrafish (*Danio rerio*): A study of bacterial diversity by using PCR-DGGE and metagenomic sequencing. PLoS ONE.

[119] Smith A.H., Mackie R.I. (2004). Effect of Condensed Tannins on Bacterial Diversity and Metabolic Activity in the Rat Gastrointestinal Tract. Appl. Environ. Microbiol..

[120] Mirpuri J., Raetz M., Sturge C.R., Wilhelm C.L., Benson A., Savani R.C., Hooper L.V., Yarovinsky F. (2013). Proteobacteria-specific IgA regulates maturation of the intestinal microbiota. Gut Microbes.

[121] Shin N.-R., Whon T.W., Bae J.-W. (2015). Proteobacteria: Microbial signature of dysbiosis in gut microbiota. Trends Biotechnol..

[122] Langille M.G.I., Zaneveld J., Caporaso J.G., McDonald D., Knights D., Reyes J.A., Clemente J.C., Burkepile D.E., Thurber R.L.V., Knight R. (2013). Predictive functional profiling of microbial communities using 16S rRNA marker gene sequences. Nat. Biotechnol..

[123] Luo C., Yi C., Ni L., Guo L. (2017). Characterization of dominant and cellulolytic bacterial communities along the gut of silver carp Hypophthalmichthys molitrix during cyanobacterial blooms. Chin. J. Oceanol. Limnol..

[124] Selma M.V., Espín J.C., Tomás-Barberán F.A. (2009). Interaction between Phenolics and Gut Microbiota: Role in Human Health. J. Agric. Food Chem..

[125] Farshkhahi F., Delazar A., Memar M.Y. (2018). Antibacterial and antibiofilm activity of grape seed extract against carbapenem resistant and biofilm producer Enterobacteriaceae. Res. J. Pharmacogn..

[126] Dhara L., Tripathi A. (2019). Cinnamaldehyde: A compound with antimicrobial and synergistic activity against ESBL-producing quinolone-resistant pathogenic Enterobacteriaceae. Eur. J. Clin. Microbiol. Infect. Dis..

[127] Zhang R., Liu L.-L., Wang X.-W., Guo C.-Y., Zhu H. (2020). Dietary tea polyphenols induce changes in immune response and intestinal microbiota in Koi carp, cryprinus carpio. Aquaculture.

[128] Hoque F., Abraham T.J., Nagesh T.S., Kamilya D. (2019). Pseudomonas aeruginosa FARP72 Offers Protection Against Aeromonas hydrophila Infection in Labeo rohita. Probiotics Antimicrob. Proteins.

[129] Nayak S. (2010). Probiotics and immunity: A fish perspective. Fish. Shellfish. Immunol..

[130] Gatesoupe F.J. (2007). Live yeasts in the gut: Natural occurrence, dietary introduction, and their effects on fish health and de-velopment. Aquaculture.

[131] Romero J., Ringø E., Merrifield D.L. (2014). The Gut Microbiota of Fish. Aquaculture Nutrition.

