# Supplementary material for: Chestnut Shell Tannins: Effects on Intestinal Inflammation and Dysbiosis in Zebrafish
Source: Animals (Basel). 2021 May 25;11(6):1538. doi: 10.3390/ani11061538 (PMC8228309; doi:10.3390/ani11061538)
Supplement: Supplementary file 1 [file animals-11-01538-s001.zip › animals-1233933-supplementary.pdf]

animals

MDPI

Supplementary Materials:

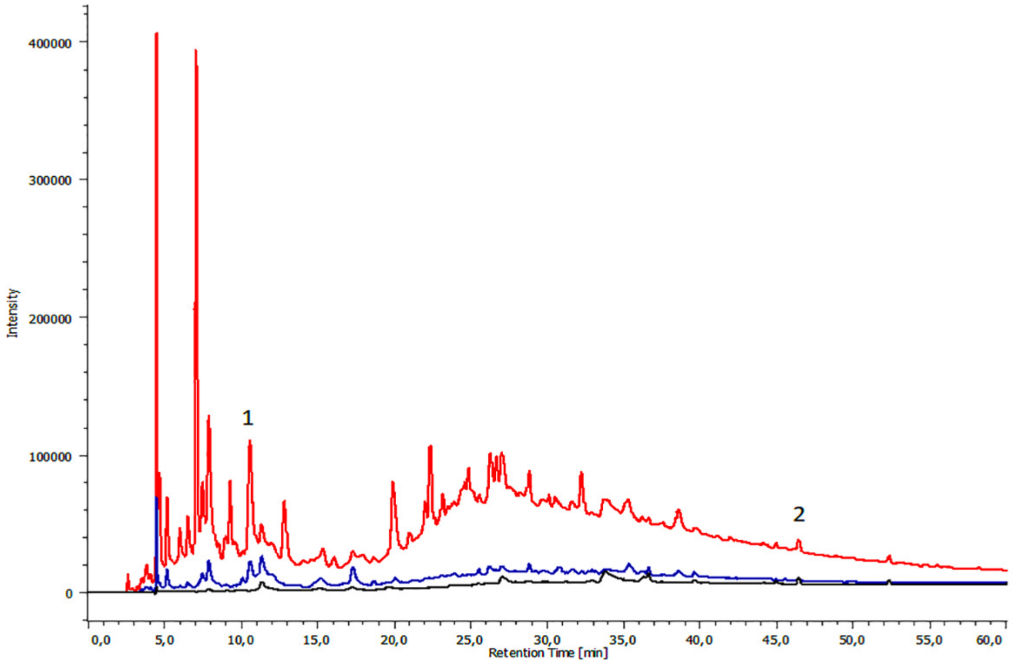

Figure S1: Chromatographic separation (HPLC/DAD) of chestnut shell extract monitoring at the wavelengths of 275 (red line), 325 (blue line), and 375 (black line) nm. The numbers indicate the following molecules: gallic acid (1), ellagic acid (2).

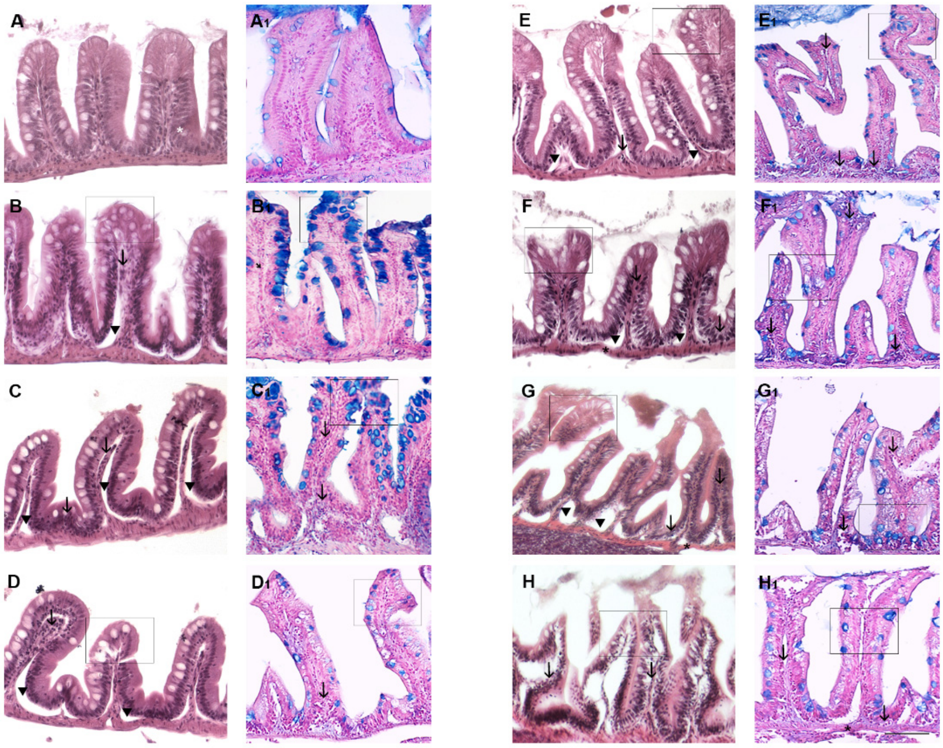

Figure S2: Hematoxylin-eosin (H&E) (**A-D**; **E-H**) and Alcian Blu (**A$_1$-D$_1$**; **E$_1$-H$_1$**) staining of MI of control zebrafish and zebrafish fed with *Artemia salina* with and without k-carrageenan. (A-

A1) MI of control zebrafish. (B-B1) MI of zebrafish fed for 3 days with 0.05% of K-carrageenan. (C-C1) MI of zebrafish fed for 3 days with 0.1% of K-carrageenan. (D-D1) MI of zebrafish fed for 10 days with 0.05% of K-carrageenan. (E-E1) MI of zebrafish fed for 10 days with 0.1% of K-carrageenan. (F-F*1*) MI of zebrafish fed for 14 days with 0.1% of K-carrageenan. (G-G1) MI of zebrafish fed for 14 days with 1% of K-carrageenan. (H-H1) MI of zebrafish fed for 28 days with 1% of K-carrageenan. Scale bar: 100 μm. Arrows indicate leucocytes infiltrates, arrowheads indicate ragged villi, asterisks indicate mucosal thinning, boxes display increased number of goblet cells.

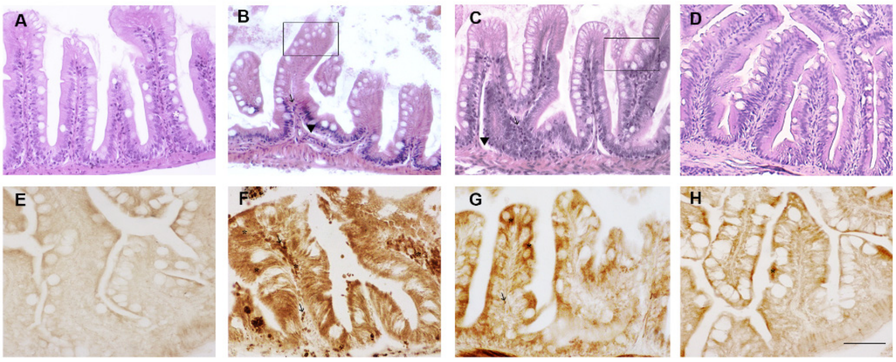

Figure S3: Haematoxylin-eosin (H&E) staining (**A-D**) and TNFα immunostaining (**E-H**) of intestine of control, inflamed (zebrafish fed for 10 days with 0.1% of K-carrageenan) and CSE (zebrafish fed with 0.4 or 4% chestnut shell extract) zebrafish. (**A**) and (**E**) Intestine of control zebrafish. (**B**) and (**F**) Intestine of inflamed zebrafish (C) and (**G**) Intestine of inflamed zebrafish followed by 0.4% of CSE. (**D**) and (**H**) Intestine of inflamed zebrafish followed by 4% of CSE. Scale bar: 100 μm for **A-D**; 50 μm for **E-H**. Arrows indicate leucocytes infiltrates, arrowheads indicate ragged villi, boxes display increased number of goblet cells, asterisks indicate TNFα immunosignal.

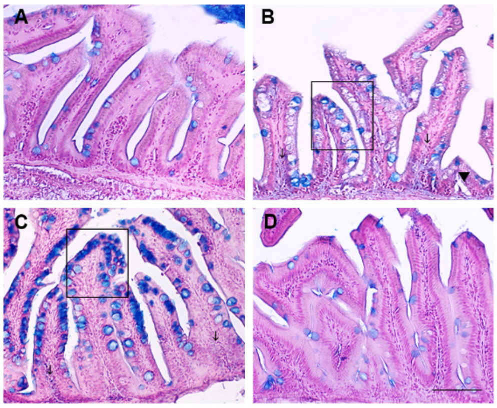

Figure S4: Alcian blu staining of intestine of (**A**) control zebrafish (C), (**B**) Inflamed zebrafish (I), (**C**) inflamed zebrafish post-treated with chestnut shell extract (CSEpostI) and (**D**) zebrafish fed with chestnut shell extract (CSE). Arrows indicate leucocytes infiltrates, arrowheads indicate ragged villi, boxes display increased number of goblet cells.

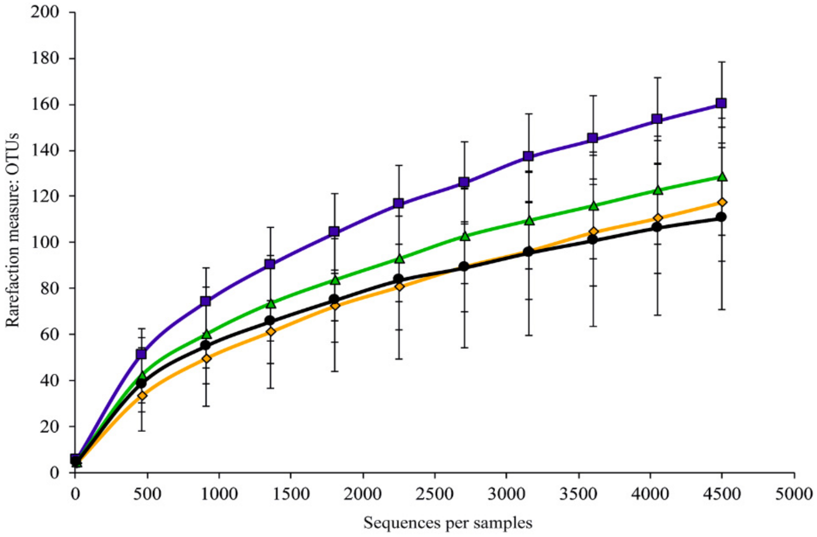

Figure S5: The rarefaction curves for microbiota community from intestine of control zebrafish (C), Inflamed zebrafish (I), inflamed zebrafish post-treated with chestnut shell extract (CSEpostI) and zebrafish fed with chestnut shell extract (CSE). ■ - C; ◇ - I; ▲ - CSE; ● - CSEpostI.

Table S1: Culturable microbiota in zebrafish (Danio rerio) fed on standard control diet and proinflammatory and polyphenolic diets.

**Microbial groups**	**Log CFU g^{-1}**			
	**C**	**I**	**CSE**	**CSEpostI**
**Total microorganisms**	7.14 ± 0.09	7.22 ± 0.01	8.78 ± 0.01	8.49 ± 0.04
**Total aerobic mesophilic bacteria**	6.95 ± 0.18	6.48 ± 0.04	8.36 ± 0.08	8.47 ± 0.04
Enterobacteriaceae	6.94 ± 0.08	6.41 ± 0.05	8.32 ± 0.04	8.34 ± 0.01
***Pseudomonas* spp.**	5.55 ± 0.01	5.16 ± 0.04	7.46 ± 0.03	5.90 ± 0.05
***Staphylococcus* spp.**	5.82 ± 0.33	5.47 ± 0.05	6.80 ± 0.10	5.98 ± 0.07
**Total anaerobic bacteria**	6.61 ± 0.09	7.09 ± 0.02	8.56 ± 0.05	7.16 ± 0.01
**Yeasts**	5.02 ± 0.02	6.06 ± 0.06	6.82 ± 0.02	6.16 ± 0.16

Results are expressed as Log of colony-forming units (CFU) per gram of analyzed intestine (Log CFU g^{-1}) and are reported as mean ± standard deviation of results obtained from triplicate analysis.

Table S2: Pairwise comparisons for richness and diversity estimates among all studied zebrafish groups (C, I, CSE, and CSEpostI) based on Kruskal–Wallis rank sum test.

Comparisons between groups	Richness estimates				Diversity estimates			
	OTU		Chao1		Simpson		Shannon	
	Z	p	Z	p	Z	p	Z	p
I vs Control	0.75	1.00	0.63	1.00	-2.77	0.017*	-2.74	0.019
I vs CSEpostI	3.60	0.001	3.62	0.0009	-0.50	1.00	-0.13	1.00
Control vs CSEpostI	2.85	0.013	2.98	0.0085	2.28	0.07	2.60	0.028
I vs CSE	0.40	1.00	0.73	1.00	-1.24	0.64	-1.26	0.62
Control vs CSE	-0.34	1.00	0.096	1.00	1.53	0.38	1.47	0.42
CSE vs CSEpostI	-3.19	0.004	-2.89	0.012	-0.75	1.00	-1.13	0.78
Kruskal–Wallis rank sum test	$\chi^2 = 16.05$, df = 3, p = 0.001		$\chi^2 = 15.63$, df = 3, p = 0.001		$\chi^2 = 8.78$, df = 3, p = 0.03		$\chi^2 = 9.65$, df = 3, p = 0.02	

The differences are significant at p ≤0.05

Table S3: Pairwise comparisons among zebrafish groups for genera with the abundance level more than 3% (One-Way PERMANOVA).

Groups	*Cetobacterium*	*Vibrio*	ZOR0006	*Shewanella*	*Flavobacterium*	*Mycoplasma*	*Aeromonas*	*Reyranella*	*Comamonas*	*Plesiomonas*	*Lysobacter*
I vs Control	0,0001/123,8*	0,0002/1,6	0,0004/23,3	0,001/20,1	0,0001/73,3	0,008/2,7	0,27/1,6	0,83/0,05	0,002/1,4	0,21/1,7	0,02/1,6
I vs CSE	0,011/8,2	0,5/0,5	0,072/3,7	0,52/0,4	0,01/6,9	0,43/1,0	0,27/0,8	0,067/3,9	0,011/4,1	0,0062/3,6	0,03/2,9
I vs CSEpostI	0,0035/12,9	0,55/0,3	0,0001/10,5	0,0006/19,7	0,0004/14,0	0,035/1,2	0,04/5,2	0,02/7,1	0,0003/3,8	0,0002/11,2	0,72/0,2
Control vs CSE	0,0002/13,7	0,0004/1,5	0,0001/36,9	0,001/17,5	0,0001/86,6	0,47/1,2	0,06/3,0	0,09/3,2	0,54/0,6	0,045/2,9	0,0004/3,0
Control vs SEpostI	0,0002/8,9	0,0001/2,3	0,0001/44,8	0,0001/41,1	0,0001/91,0	0,0005/1,2	0,009/8,6	0,013/6,1	0,51/0,3	0,0012/10,2	0,14/2,1
CSE vs CSEpostI	0,56/0,3	0,22/1,3	0,016/5,2	0,085/3,3	0,03/5,4	0,005/1,2	0,33/1,2	0,47/0,5	0,77/0,1	0,20/1,8	0,01/3,0
Total sum of squares	29760	4121	9259	1257	1185	1017	2765	432,6	2270	21610	3940
Within-group sum of squares	13500	3761	2597	486,3	152,9	925,9	2147	336,5	2050	14680	3161
F	14,45	1,15	30,79	19,03	81,04	1,19	3,46	3,42	1,29	5,67	2,96
p (same)	0,0001	0,32	0,0001	0,0001	0,0001	0,0017	0,033	0,027	0,29	0,0028	0,0015

* p/F; the differences are significant at $p \leq 0.05$; The number of permutations was 9999.

Table S4: Thirtythree shared bacterial taxons among zebrafish groups of intestinal content (core microbiome)

Phylum	Family	Genus
Proteobacteria	*Aeromonadaceae*	*Aeromonas* (8)*
	Beijerinckiaceae	*Bosea*
	Burkholderiaceae	*Comamonas Comamonas aquatic*
		Cupriavidus
		Delftia
		Diaphorobacter
		NI**
	Desulfovibrionaceae	NI
	Enterobacteriaceae	*Plesiomonas*
	Moraxellaceae	*Acinetobacter* (2)
		Enhydrobacter
	Pseudomonadaceae	*Pseudomonas*
	Reyranellaceae	*Reyranella* (4)
	Rhodobacteraceae	NI
Actinobacteria	*Nocardiaceae*	*Rhodococcus*
Acidobacteria	NI	NI
Firmicutes	*Erysipelotrichaceae*	ZOR0006
Planctomycetes	*Pirellulaceae*	NI
Fusobacteria	*Fusobacteriaceae*	*Cetobacterium*
Bacteroidetes	*Flavobacteriaceae*	*Flavobacterium*
Verrucomicrobia	*Pedosphaeraceae* (2)	NI

* – the number in parentages is the quantity of different OTUs belonging to the genus; **NI – the taxon has not identified
